# Prolonged systemic inflammation worsens impairments to astrocyte Ca^2+^ and functional hyperemia in Alzheimer's disease

**DOI:** 10.1002/alz.71607

**Published:** 2026-07-23

**Authors:** Chang Liu, Kimia Sakha, Jaime Anton, Alfredo Cardenas‐Rivera, Mohammad A. Yaseen

**Affiliations:** ^1^ Department of Bioengineering Northeastern University Boston Massachusetts USA; ^2^ Department of Biomedical Engineering Southern University of Science and Technology Shenzhen Guangdong China

**Keywords:** Alzheimer's disease, astrocyte Ca2^+^, lipopolysaccharide, neuroinflammation, neurovascular coupling, two‐photon microscopy

## Abstract

**INTRODUCTION:**

Chronic neuroinflammation in Alzheimer's disease (AD) alters astrocyte physiology and neurovascular unit function. AD patients frequently experience recurrent systemic inflammatory insults from comorbid conditions, which act as “secondary‐hits” believed to worsen cognitive decline. The impact of these secondary insults  on astrocyte‐mediated neurovascular regulation remains unknown.

**METHODS:**

We applied intravital two‐photon microscopy to longitudinally investigate astrocytic Ca^2^^+^ dynamics and functional hyperemia during sensory stimulation in APP/PS1dE9 mice before and during secondary lipopolysaccharide (LPS)‐induced systemic inflammation.

**RESULTS:**

AD mice exhibited diminished stimulation‐evoked astrocytic Ca^2^^+^ activity, while functional hyperemia remained largely preserved. LPS further suppressed astrocytic Ca^2^^+^ responses and produced temporally specific vascular alterations, with AD and wild‐type mice following divergent inflammatory trajectories.

**DISCUSSION:**

Our findings provide the first in vivo longitudinal characterization of how secondary systemic inflammation disrupts astrocyte‐mediated neurovascular regulation. The selective vulnerability of astrocytic Ca^2^^+^ signaling relative to vascular output implicates recurrent inflammatory insults as a clinically relevant contributor to neurovascular dysfunction in preclinical AD.

## Highlights

- In the APP/PS1dE9 mouse model of Aβ pathology, astrocytic Ca^2^^+^ signaling is selectively attenuated during sustained sensory stimulation at age 12 to 13 months, while functional hyperemia at the arteriole and capillary levels remains largely unchanged.
- A 14‐day period of LPS‐induced systemic inflammation, modeling a secondary systemic inflammatory threat, further suppresses astrocytic Ca^2^^+^ responses across compartments and produces temporally specific vascular alterations, with AD and WT mice following divergent inflammatory trajectories.
- Linear mixed‐effects modeling reveals that AD mice exhibit delayed but progressive astrocytic Ca^2^^+^ suppression and selective late‐phase vascular vulnerability, while WT mice show earlier and more pronounced Ca^2^^+^ suppression with comparatively preserved vascular responses.
- Prolonged systemic inflammation reduces Aβ deposition in the AD brain, yet concurrently impairs astrocyte physiology and neurovascular signaling, highlighting a dissociation between amyloid burden and astrocyte‐mediated physiological support.

## 1 BACKGROUND

Vascular dysfunction and neuroinflammation are two major pathological features of preclinical Alzheimer's disease (AD) and other diseases of the central nervous system (CNS).^1^, ^2^, ^3^, ^4^ These notable AD hallmarks are coupled through their shared cellular components, with astrocytes performing especially critical roles in regulating both microvascular blood flow and brain immunity.^5^, ^6^ Astrocyte excitability is mediated primarily by Ca^2+^ signaling and is crucial for numerous immune, synaptic, metabolic, and hemodynamic signaling pathways of the “neuro‐glio‐vascular unit” (NGVU).^7^, ^8^, ^9^ In response to stress, injury, or disease‐related alterations, astrocytes undergo phenotypic and morphological changes to a reactive state. Reactive astrocytes demonstrate altered excitability, dysregulated Ca^2+^ signaling, altered gene expression, metabolic shifts, and active release pro‐ or anti‐inflammatory cytokines, and they contribute notably to AD progression and other CNS disorders.^7^, ^10^, ^11^ In AD and other disorders, reactive astrocytes reportedly facilitate amyloid beta (Aβ) pathology and neural network dysfunction.^12^, ^13^ The impact of inflammatory astrocyte phenotype alterations on their contributions to cerebral blood flow (CBF) regulation is presently unclear.

Neuroinflammation reportedly potentiates Aβ accumulation, cerebrovascular pathology, mitochondrial dysfunction, reactive oxygen species generation, and synaptic loss. Thus, it is increasingly recognized as a driving force and critical accelerator for the aggressive cycle of pathological deteriorations in preclinical AD and other neurodegenerative disorders.^14^, ^15^ Many investigators assert a nuanced influence of neuroinflammation, citing evidence of both harmful and beneficial effects of an inflammatory stimulus during disease progression.^16^, ^17^ Several studies also highlight the importance of the brain's initial inflammatory state on subsequent outcomes to a secondary inflammatory threat. At early stages of AD or other neurodegenerative diseases, acute periods of illness or systemic inflammation can catalyze chronic neuroinflammation and exacerbate neurotoxicity and functional decline.^18^, ^19^, ^20^, ^21^ Recent evidence suggests that this vulnerability is compounded by inflammaging – the age‐related accumulation of pro‐inflammatory mediators that progressively primes both peripheral and central immune compartments, lowering the threshold for neuroinflammatory responses to subsequent insults.^22^ Under this framework, systemic inflammatory exposures arising from comorbid conditions such as infections, metabolic disorders, or cardiovascular disease may act not merely as acute triggers but as cumulative contributors to immune priming that accelerates AD pathology.^22^ Collectively, these views motivate investigations to explore the effects of enhancing or repressing a pro‐inflammatory response on chronic cerebral pathologies.^23^

**RESEARCH IN CONTEXT**

1. **Systematic review**: Astrocytes are essential regulators of CBF and are chronically activated in the AD brain. AD patients frequently experience recurrent systemic inflammatory insults from comorbid conditions, further aggravating a pre‐existing neuroinflammatory environment. Epidemiological evidence implicates such secondary inflammatory insults as accelerators of cognitive decline. The impact of this “second‐hit” burden on astrocyte‐mediated neurovascular regulation in the AD brain is currently unknown.
2. **Interpretation**: Using longitudinal intravital two‐photon microscopy in awake APP/PS1dE9 mice, we demonstrate that AD is associated with attenuated sustained astrocytic Ca^2^^+^ responses during prolonged sensory stimulation, while functional hyperemia remains largely unaltered. A 14‐day secondary inflammatory challenge induced by LPS further suppressed astrocytic Ca^2^^+^ signaling and provoked temporally specific vascular alterations. Critically, AD and WT mice followed divergent inflammatory trajectories rather than showing uniform impairment.
3. **Future directions**: This study establishes the first in vivo longitudinal framework for understanding how secondary systemic inflammation disrupts astrocyte‐mediated neurovascular function in AD. Future investigations can extend knowledge of the mechanistic underpinnings by directly interrogating molecular mediators of astrocytic Ca^2^^+^ dysregulation and determine whether restoring astrocyte Ca^2^^+^ dynamics is sufficient to preserve neurovascular coupling under combined amyloid and inflammatory stress.

Previous studies also showed that acute systemic inflammation disrupted cerebral hemodynamics in healthy rodent brains and human patients with small vessel disease and traumatic brain injury.^24^, ^25^, ^26^ To precisely elucidate the intricate relationship between neuroinflammation and vascular dysfunction in preclinical AD, a detailed understanding of neuroinflammation's impact on astrocyte Ca^2+^‐mediated microvascular hemodynamics is essential. In this study, we investigated the effects of amyloid‐induced inflammation coupled with a prolonged systemic inflammatory threat on astrocyte Ca^2+^ signaling and CBF using a second‐hit paradigm designed to examine exacerbation of pre‐existing pathology under clinically relevant inflammatory conditions. Our approach is motivated by emerging evidence that inflammaging and peripheral immune priming represent central mechanisms through which lifelong systemic inflammatory exposures interact with and accelerate AD pathology.^22^ We specifically aimed to examine how a brain with advanced amyloid pathology and pronounced pre‐existing neuroinflammation responds to a secondary systemic inflammatory challenge. At 12 to 13 months, amyloid burden, reactive astrogliosis, and neurovascular dysfunction are more severe than at earlier disease stages in this model, providing a more vulnerable baseline for detecting the effects of secondary systemic inflammation. The current study builds upon our prior observations in 8‐month‐old APP/PS1dE9 mice, where LPS‐induced inflammatory effects on cerebral oxygen extraction were already more pronounced in AD than in WT mice despite only modest baseline neuroinflammatory differences.^27^ APPswe/PS1dE9 mice and their wild‐type (WT) littermates were exposed to a 14‐day period of systemic inflammation induced by i.p. injection of the bacteria‐derived endotoxin lipopolysaccharide (LPS).^28^ Using intravital two‐photon microscopy, we longitudinally measured Ca^2+^ release from astrocytes and microvascular hemodynamics in penetrating arterioles and capillary branches under resting‐state conditions and during functional brain activation in the awake mouse brain. Our observations demonstrate that Aβ‐induced neuroinflammation selectively attenuates sustained astrocytic Ca^2^^+^ responses while largely preserving functional hyperemia. A 14‐day secondary inflammatory challenge further suppressed astrocytic Ca^2^^+^ signaling across compartments and produced temporally specific vascular alterations, with AD and WT mice following divergent inflammatory trajectories, with AD mice showing delayed but progressive astrocytic suppression and selective late‐phase vascular vulnerability and WT mice exhibiting earlier and more pronounced Ca^2^^+^ suppression with comparatively preserved vascular responses. Together, these results delineate the interdependent relationships between astrocytic Ca^2^^+^ dysregulation, neuroimmune priming, and neurovascular vulnerability during the multifactorial progression of preclinical AD.

## 2 METHODS

### 2.1 Animal preparation

All experiments were performed following ARRIVE guidelines for animal care, under a protocol approved by the Northeastern University Institutional Animal Care and Use Committee. The mouse strain used for this research project, B6C3‐Tg(APPswe, PSEN1dE9)85Dbo/Mmjax, RRID:MMRRC_034829‐JAX, was obtained from the Mutant Mouse Resource and Research Center (MMRRC) at The Jackson Laboratory, a National Institutes of Health (NIH)‐funded strain repository, and was donated to the MMRRC by David Borchelt, Ph.D., McKnight Brain Institute, University of Florida. The double transgenic AD mouse co‐expresses the Swedish mutation of amyloid precursor protein (APP) and a mutant human presenilin 1 gene (PS1‐dE9) and accumulates Aβ deposits in the cortex at 6 months.^29^ The study used 12‐ to 13‐month‐old female APP/PS1dE9 mice and their WT littermates (*n* = 8 per cohort). Female APPswe/PS1dE9 mice develop more severe amyloid deposition and exhibit a more pronounced neuroinflammatory baseline, including increased cytokine levels, astrogliosis, and microgliosis, compared to males.^30^, ^31^ This “primed” inflammatory state makes female mice a suitable model for investigating the impact of a secondary inflammatory challenge within the framework of the “second‐hit” paradigm.

At 10 months of age, mice underwent cranial window surgery followed by glial fibrillary acidic protein (GFAP)‐promoted GCaMP6f virus injection. Under isoflurane anesthesia (3% to 3.5% isoflurane for induction and 1% to 2% for maintenance), hair and skin were removed to expose the skull, and a titanium butterfly‐shaped head post was implanted using dental cement.^32^ After the localization of the injection site, an ∼3‐mm‐diameter cranial window was created on the barrel cortex of the mouse, followed by targeted injection of a GFAP‐promoted GCaMP6f virus (pZac2.1gfaABC1D‐cyto‐GCaMP6f, Plasmid #52925, Addgene). Virus (300 to 500 nL) was injected at 2 nL/s, 300 to 350 µm below the brain surface, using a glass micropipette and a Nanoinjector III (Drummond Scientific).^33^ Injections were performed in two to three cortical regions to increase the area of viral expression. The procedure lasted 15 to 20 min, with the dura kept hydrated throughout. After the injections, the dura was covered with a CNC‐machined transparent acrylic coverslip (outer diameter: 5 mm, inner diameter: 3 mm). The mouse was then removed from the stereotaxic frame and allowed to recover from anesthesia. The animal was single‐housed and provided with 5 days of post‐operative care (40 mg/mL sulfamethoxazole [SMX] and 8 mg/mL trimethoprim [TMP] in drinking water). Buprenorphine (0.05 mg/kg at 0.03 mg/mL) was administered subcutaneously for 3 days after surgery. To label astrocytes in the activation center, we conducted optical intrinsic signal imaging (OISI) and whisker stimulation on the anesthetized mouse before removing the skull. The skull over the barrel cortex (A‐P: 2 mm, M‐L: 3 mm) was thinned, and whiskers on the contralateral side of the thinned skull were deflected using a cotton swab. The skull was illuminated with an LED light source during the stimulation through a green bandpass filter (568/10 nm). The reflected light was recorded with a CMOS camera (Basler acA1300–200 um, Edmund optics) mounted on the surgical microscope, and the relative intensity change was calculated with a customized MATLAB script. Regions with the greatest intensity drop were identified as the activation area and selected as the site for viral injection.

### 2.2 Habituation training

Headpost restraint training started 7 to 10 days after surgery using a custom‐made imaging cradle. During training, the mouse was conditioned to tolerate head immobilization from 5 min to 1 h. Whisker stimulation was included during the final training sessions to acclimate the animal to the functional activation experiment. Milk was given as a reward during the training.

### 2.3 Systemic inflammation induction

Peripheral inflammation induced by systemic injection of LPS has been shown to transfer to the CNS and cause a prolonged inflammatory response in the brain.^34^ Systemic inflammation was induced through daily i.p. injections of 0.3 mg/kg LPS (*E. coli* O55: B5, L4005, CAS‐No. 93572‐42‐0, Sigma‐Aldrich) for 14 days. A stock solution of LPS was prepared at 1 mg/mL in phosphate‐buffered saline (PBS) and stored at −20°C. The solution was vortexed and diluted four‐fold to 0.25 mg/mL for each injection.

### 2.4 Functional activation protocol

A capillary tube with a three‐dimensional (3D)‐printed broom‐like end was positioned approximately 2.5 to 3 cm from the nose, parallel to the right side of the mouse's face, and below the eye to avoid directing air toward the eyes during stimulation. Air puffs were delivered through the tube and controlled using a pneumatic drug injection system (PDES‐DXH, ALA Scientific, USA). Two whisker stimulation protocols – brief and prolonged – were performed. The brief protocol consisted of a 5‐s baseline, 2.7 s of 1.67 Hz stimulation (5 pulses, 20 psi), and 22.3 s of recovery. The prolonged protocol included a 5‐s baseline, 30 s of 1.67 Hz stimulation (50 pulses, 20 psi), and 30 s of recovery. Short and long stimulations were interleaved within each trial and repeated 10 times.

### 2.5 Functional activation center mapping

The activation center of whisker stimulation was mapped using OISI under the brief whisker stimulation protocol. A white LED illuminator (SCHOTT KL 1600) with a 568/10 nm bandpass filter (Edmund Optics) served as the light source. The camera was triggered by a TTL signal from a general‐purpose input/output (GPIO) hardware trigger. A fiber‐optic LED light source illuminator (SCHOTT KL 1600 LED) delivered light to the cranial window at a 60° angle. Reflected light was collected using a 4× objective (Nikon Instruments Inc.) and recorded at 5 Hz for 20 s with a CMOS camera (Basler acA1300–200u m , Edmund Optics). Baseline intensity (I_0_) was calculated by averaging the intensity over the baseline recording. Relative intensity (I/I_0_) was calculated for all imaging frames. Cortical area with the maximal intensity decreases was identified as the activation center.

### 2.6 Two‐photon imaging in awake mice

Two‐photon imaging of astrocyte Ca^2+^ signaling and vascular dynamics at the barrel cortex in resting state and during functional activation was conducted using the Ultima2pPlus multiphoton imaging system (Bruker Nano, Inc.) with a 25× objective (1.10 NA, Nikon Instruments Inc.). Imaging during the functional activation experiment was synchronized with the whisker stimulation protocol. Blood vessels were labeled through retro‐orbital injection of Texas Red Dextran (2.5% w/v, 80 µL). A tunable ultrafast laser (InSight X3+, Spectral‐Physics) was tuned to 940 nm for simultaneous imaging of blood vessels and astrocytic Ca^2+^ imaging. We tracked the penetrating arterioles from the pial surface to the deepest assessable cortical layer and recorded the vessel diameter and the astrocytic Ca^2+^ release using a minimal frame rate of 4.3 Hz. The image field of view (FOV) was around 88 × 88 µm^2^. On the following day, capillary diameter and red blood cell (RBC) velocity in response to the interleaved functional activation was measured with two‐photon line scanning,^35^ with a scan frequency ranging from 369.87 to 1034.8 Hz. Penetrating arterioles perpendicular to the image plane and capillaries parallel to it were selected to ensure accurate diameter estimation, while tilted vessels were excluded during post‐processing. We measured regions from 49 to 414 µm below the pial surface for WT mice and 30 to 360 µm below the pial surface for AD mice.

### 2.7 Two‐photon imaging data analysis

#### 2.7.1 Astrocyte spontaneous Ca^2+^

Image frames were pre‐processed using a non‐rigid motion correction algorithm,^36^ followed by median filtering. Resting‐state astrocyte Ca^2+^ fluctuations were quantified using a region of interest (ROI)‐based Ca^2+^ analysis tool: “GECIquant” in ImageJ.^37^ Briefly, average intensity projection was generated from the time series of two‐photon raster scan images. We created a binary ROI mask where the area within the polygon ROI was set to 1 and the pixels in the rest of the image were set to 0. Mean intensity within the ROI over time was calculated. The area criterion was set to 30 µm^2^ to Inf for astrocyte soma and endfeet, and 8 to 2000 µm^2^ for Ca^2+^ waves in processes. Relative intensity change ($\Delta F/\;F_{0\;}=\left(F_{t}\;-F_{0\;}\right)/F_{0\;})$ was calculated. $F_{0}$ was calculated as the median of the lowest 5% fluorescence intensity over the entire 10‐min raster scan. $\Delta F/F_{0}$ of all astrocyte ROIs from all mice were interpolated with a dt=0.2244s (i.e., 4.45 fps). The $\Delta F/F_{0\;}$ traces were pre‐processed using linear detrending to remove intensity drift that occurred during the measurement. The detrended intensity traces were then low‐pass filtered with a cutoff frequency of 0.1 Hz, as determined by power spectrum analysis. To determine the threshold for the Ca^2+^ event, we sorted the normalized intensity values in ascending order, calculated the median absolute deviation (MAD) of the lowest 80% of the ΔF/F_0_ values, and used five times the MAD as the threshold for the Ca^2+^ event. This threshold is robust to outliers and helps to reduce the number of false positives. Ca^2+^ events were detected and quantified using the “findpeaks” function in MATLAB. The detected events were categorized as either “singlepeak” or “multipeak” events as described.^38^ We calculated the frequency of the event as per minute per ROI.

#### 2.7.2 Arteriole diameter

Arteriole diameter in response to functional activation was quantified using the automatic structure tracking algorithm developed by Haidey et al.^39^ The algorithm tracks the cross‐sectional area of the vessel based on user‐defined luminal edges. Vessel diameter (D) was calculated from the cross‐sectional area. Relative change of diameter ($\Delta D/\;D_{0\;}=\left(D_{t}\;-D_{0\;}\right)/D_{0\;}\times 100)$ was calculated, where the baseline diameter ($D_{0}$) was the average diameter over the 5‐s resting‐state measurement. Since the frame rates of different imaging fields sometimes vary slightly, $\Delta D/D_{0}$ were interpolated with Δt=0.2244s.

#### 2.7.3 Quantifying stimulus‐evoked responses

To quantify stimulus‐evoked arteriole dilation and astrocyte Ca^2+^ release, we first scrutinized all 10 stimulation trials and excluded those with motion artifacts. The remaining trials were classified as either “with response” or “without response.” Trials exhibiting responses were averaged to determine the mean ΔD/D_0_ (arteriole dilation) and ΔF/F_0_ (Ca^2+^ activity) for each ROI. The averaged arteriole dilation and Ca^2+^ signals were low‐pass filtered with a cutoff frequency of 0.2 Hz. Peak responses were identified using the “findpeaks” function in MATLAB. For each detected peak, the amplitude and latency were calculated. Peak latency was defined as the time interval between stimulus onset and the peak response. The area under the curve (AUC) was calculated for each stimulation paradigm, as reported in a previous publication.^40^ For 3‐s whisker stimulation, AUC was computed over the 0‐ to 25‐s window, while for 30‐s stimulation, it was assessed over 0 to 60 s. Additionally, for 30‐s stimulation, the average response during the last 2 s of stimulation was calculated to assess late‐phase activity. To analyze response kinetics, we followed the method used in a recent publication to fit ΔD/D_0_ and ΔF/F_0_ traces with a sigmoidal model.^41^ The rise part of the trace was fitted using the equation $f\;\left(x\right)=\frac{a}{1+e^{-b(x-c)}}$, and the decay part of the trace was fitted using the four‐parameter logistic equation $f\;\left(x\right)=d+\frac{a-d}{1+{\left(\frac{x}{c}\right)}^{b}}$. Rise time was calculated as the interval between 10% and 90% of the fitted peak response amplitude. Duration was calculated as the full width at half maximum (FWHM). Note that responses to 30‐s stimulation have a plateau phase, and the decay part of the traces was defined when the responsive trace start to return to the baseline. The duration for 30‐s stimulation is determined as the time interval from the half‐maximal of the rising phase to the half‐maximal of the decay phase.

#### 2.7.4 Capillary diameter

Space–time images from two‐photon line scans were denoised using a 2D median filter. Vessel diameter was calculated using the MATLAB two‐photon imaging analysis toolbox, CHIPS.^42^ The algorithm normalizes the vessel's intensity profile to a range of 0 to 1 and calculates the diameter using the full width at a user‐defined height. We used the full width at 0.4 of the maximum intensity, as it provided the most robust diameter estimation based on our signal‐to‐background ratio. The sampling rate was set at a maximum of 20 Hz. Measurements affected by motion, particularly along the axial axis, were discarded. Average diameter and ΔD/D_0_ across all selected stimulation trials were calculated. The ΔD/D_0_ traces were smoothed using a moving median filter with a 1‐s window (20 data points). Vascular dilation amplitude, AUC, and kinetics were analyzed using the same method as in raster‐scan data analysis. Vessels were categorized by branch order: penetrating arterioles (0th order), lower‐order capillaries (1st and 2nd order), and higher‐order capillaries (> = 3rd‐order branches). Resting‐state capillary diameter was calculated by averaging the baseline measurement taken from 1.5 to 4.5 s.

#### 2.7.5 Capillary RBC velocity

The space‐time images were filtered using Sobel filtering, and the capillary RBC velocity was analyzed using the iterative Radon Transform.^43^ Image segments of every 100 lines were used to calculate RBC velocity. Twenty‐five lines were skipped before calculating the velocity at the next time point, leading to a time window of the RBC velocity of 24  to 67 ms. Relative velocity (Δv/v_0_,%) was calculated and filtered with a low‐pass filter with a cutoff frequency of 0.1 Hz. All Δv/v_0_ traces were interpolated with dt = 0.05 s.

#### 2.7.6 Line scan measurement of astrocyte Ca^2^

Line segments measuring the astrocyte processes and endfoot wrapping around the capillaries were selected during post‐processing. Total intensity along the line was calculated and normalized to ΔF/F_0_ (%). The normalized intensity traces were also downsampled to 20 Hz. Amplitude, AUC, and kinetics were quantified.

### 2.8 Ex vivo immunofluorescence (IF) imaging

After 14 days of LPS treatment, mice were sacrificed, and brains were collected for IF staining. IF staining was performed on a subset of the imaged mice (*n* = 3 per cohort). Mice received 14 days of saline injections at a volume equivalent to the LPS dose of 0.3 mg/kg served as control group (*n* = 2 for WT, *n* = 3 for AD). A separate group of mice received 7 days of LPS injection was used as well (*n* = 2 for AD, *n* = 3 for WT). Mice were intracardially perfused with 40 mL of heparin‐PBS solution, followed by 20 mL of 4% paraformaldehyde (PFA) for fixation. The brains were collected and stored in 4% PFA for a maximum of 24 h, then transferred to 70% ethanol and stored in a $-4^{\circ }C$ fridge. The brain samples were sent to the Dana‐Farber/Harvard Cancer Center Specialized Histopathology Services Core (Boston, MA, USA) for IF staining. The samples were paraffin‐embedded and sliced into approximately 10‐mm‐thick sections for IF staining. Nuclei were stained with DAPI, microglia with an anti‐Iba‐1 antibody labeled with Alexa Fluor 594, and astrocytes with an anti‐GFAP antibody labeled with Alexa Fluor 647. IF images were collected with a confocal microscope (LSM 800, ZEISS). DAPI, AF‐647, and AF‐594 were excited with 405, 640, and 561 nm, respectively. Images were taken using 5 × and 10 × objectives. Barrel cortex and hippocampus were identified using the Allen Brain Atlas. For each 10 × FOV ($638\;\times \;638\;um^{2}$), Z‐stack images were collected with a step size of 0.34 µm. All images were acquired using consistent excitation power and emission detector gain settings. A β_1‐42_ was stained with anti‐A β_1‐42_ antibody conjugated to Alexa Fluor 647. Fluorescence images of A β_1‐42_ were acquired with wide‐field fluorescence microscopy (Zeiss Axio Observer). All images were acquired using the same exposure time.

GFAP and Iba‐1 expression were quantified using the maximum intensity projection (MIP) of the z‐series images. Background was removed and MIP images were binarized using an intensity threshold. The percentage area of pixels with intensity above the intensity threshold was calculated. Similarly, percentage area of A β_1‐42_ load in cortex and hippocampus was quantified in ImageJ.

### 2.9 Statistical analysis

Statistical analyses were performed in R (RStudio) using linear mixed‐effects models (LMM; lme4 package). Differences in spontaneous astrocyte Ca^2+^ dynamics, stimulus‐evoked vessel dilation, and astrocyte Ca^2+^ responses between WT and AD mice were assessed with genotype as a fixed effect and animal as a random effect. To evaluate the effects of LPS in WT and AD mice, and to assess whether the two genotypes responded differently to LPS, time (days 0, 7, and 14) and genotype were included as fixed effects, with animal as a random effect. Pairwise comparisons were performed using Tukey's post hoc test (emmeans package). Significance levels were set as follows: *p < 0.05, **p** < 0.01**, ***p < 0.001, and ****p < 0.0001.

## 3 RESULTS

Among its many functions, astrocyte Ca^2+^ is an important modulator of basal blood flow and functional hyperemia in the brain.^6^, ^44^ To determine whether Aβ‐induced inflammatory astrogliosis altered astrocyte Ca^2+^‐mediated contributions to CBF regulation, we measured astrocyte Ca^2+^ dynamics and microvascular dilation under resting‐state conditions and during sensory‐evoked functional hyperemia in the awake mouse brain at age 12 to 13 months in APPswe/PS1dE9 and WT mice. We then explored whether a 14‐day LPS‐induced neuroinflammation challenge alters astrocyte calcium signaling and its contributions to neurovascular coupling. Figure S1 illustrates the timeline for the experimental procedure.

Recent studies demonstrate that astrocyte Ca^2+^ contributes significantly to sustaining functional hyperemia in response to prolonged, but not brief, periods of cortical stimulation.^40^ Motivated by these findings, we used two‐photon imaging to measure brain hemodynamics and astrocytic Ca^2+^ release in response to both brief (3 s) and sustained (30 s) whisker stimulation. Intravital two‐photon microscopy measurements were performed in cortical layers I and IV of awake mice. Data from both layers were combined.

### 3.1 Pre‐inflammation: reduced stimulus‐evoked astrocyte Ca^2+^ in AD mice

**FIGURE 1:**
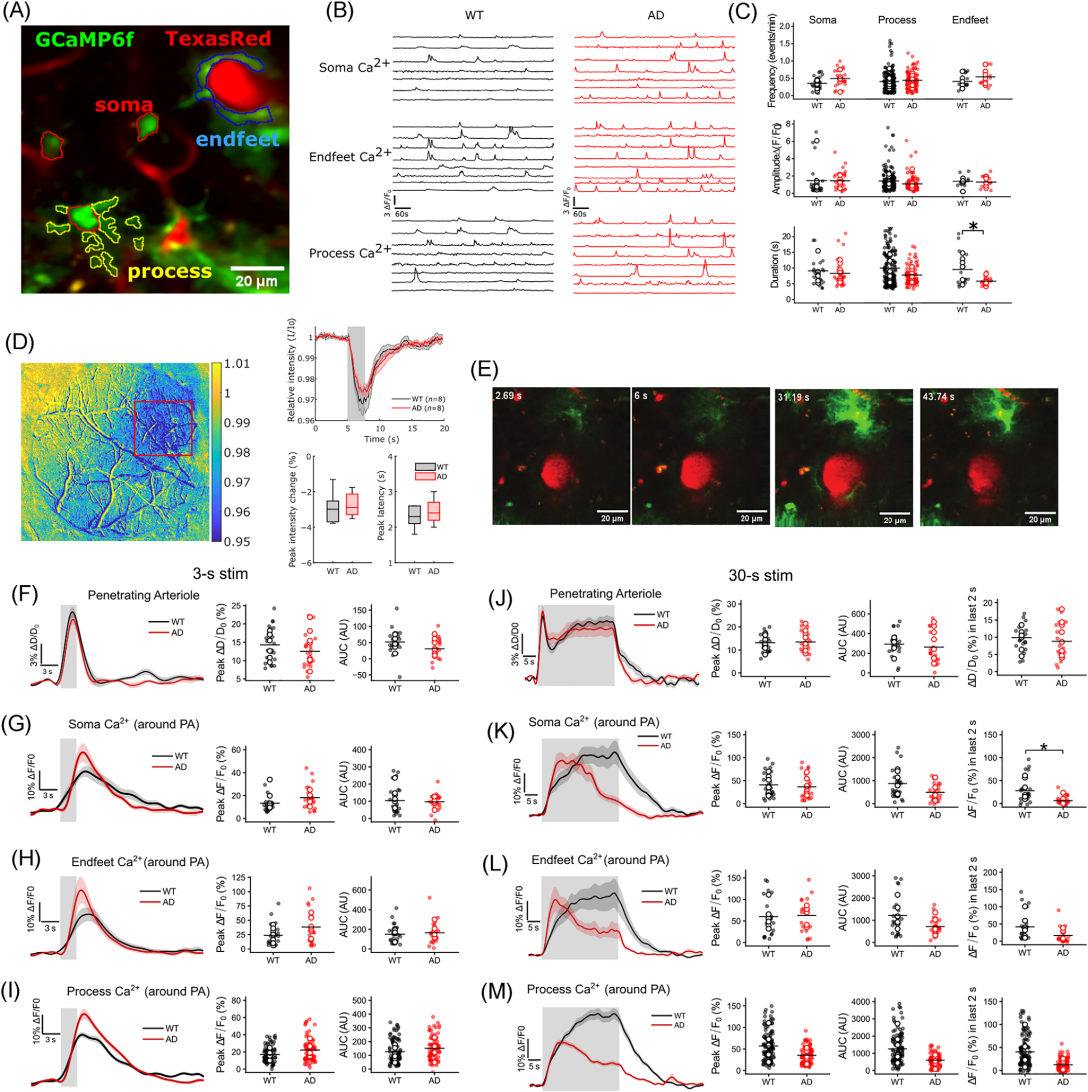
Baseline (pre‐LPS) astrocyte Ca^2+^ activity and sensory‐evoked neurovascular responses in AD and WT mice. (A) Two‐photon raster scan image (average intensity projection over 10 min) showing vasculature labeled with Texas Red and astrocyte microdomains expressing GCaMP6f. Annotated regions include soma (red), endfeet (blue), and process (yellow). (B) Representative traces of spontaneous Ca^2+^ activity recorded from astrocyte soma, endfeet, and processes over 10 min in WT (black) and AD (red) mice under baseline (pre‐LPS) conditions. (C) Amplitude, frequency, and duration of Ca^2+^ spikes at different astrocyte ROIs. Sample sizes are summarized in Table S1. (D) Optical intrinsic signal imaging (OISI) of cerebral hemodynamic responses to 3‐s whisker stimulation in the left barrel cortex. (E) Representative two‐photon images showing penetrating arteriole dilation and astrocyte Ca^2^^+^ responses during a 30‐s whisker stimulation trial at baseline. (F–I) Trial‐averaged time courses and quantification of penetrating arteriole dilation and astrocyte Ca^2^^+^ responses (soma, endfeet, and processes) evoked by 3‐s whisker stimulation. (J–M) Trial‐averaged time courses and quantification of penetrating arteriole dilation and astrocyte Ca^2^^+^ responses (soma, endfeet, and processes) evoked by 30‐s whisker stimulation. Time courses were obtained by averaging responses across trials for each ROI, followed by averaging across ROIs. Shaded areas indicate the SEM across ROIs, calculated from trial‐averaged responses for each ROI. Small dots represent individual ROIs, larger dots indicate per‐animal means, and horizontal lines denote the group mean across animals. Statistical analyses were performed using a linear mixed‐effects model (genotype as a fixed effect; mouse as a random effect).*p < 0.05, **p < 0.01, ***p < 0.001.

We first evaluated resting‐state spontaneous cortical astrocyte Ca^2+^ by imaging GCaMP6f‐labeled astrocytes in awake mice. Raster scanning was performed in ∼88 µm^2^ cortical regions surrounding penetrating arterioles for 10 min at ∼4.4 Hz (Figure  1A). Dynamic Ca^2+^ transients were quantified and analyzed from the soma, processes, and endfoot compartments of astrocytes. In AD mice, we observed ∼15% to 40% higher frequencies of Ca^2+^ spikes compared to age‐matched WT littermates, yet the difference did not reach statistical significance (Figure  1B,C, left, *p* = 0.067 soma). No differences in the Ca^2+^ amplitudes were observed between AD and WT mice. Compared to WT littermates, the mean duration of the astrocytic Ca^2+^ spikes in AD mice was significantly lower (38.95%, *p* = 0.048) in astrocyte endfeet. Shorter Ca^2+^ durations were also observed in the soma and endfoot compartments in AD mice; however, the differences did not reach statistical significance (Fig.  1C).

Next, we investigated whether amyloid pathology impacted functional hyperemia. We first applied OISI during a 3‐s whisker stimulus to locate the activation center (Figure 1D). We measured astrocyte Ca^2+^ dynamics and diameter of penetrating arterioles while stimulating the mouse barrel cortex via pneumatic whisker deflection. Our OISI measurement revealed slight, but not significant, differences in peak cerebral blood volume response (< −1%) in AD mice relative to WT (Figure 1D). The activated region was then imaged via TPM using both raster scanning (4.4 Hz, Figure 1E) and rapid line scanning (∼360 to 1000 Hz, downsampled to 20 Hz during data processing).

In response to a short ∼3‐s functional stimulus, AD and WT mice demonstrated comparable responses for arterial dilation and Ca^2+^ signaling within astrocyte compartments. For arterial dilation response, the AUC, arterial peak dilation, rise times, and latency were similar between cohorts (Figures 1F and S2A). Astrocyte Ca^2+^ kinetics varied in different astrocytic microdomains, as reported previously.^45^ Peak amplitudes of stimulus‐induced Ca^2+^ release in AD mice appeared higher in all astrocyte compartments compared to WT mice; however, the differences did not achieve statistical significance (Figure 1 G–I). AD mice demonstrated significantly shorter Ca^2+^ responses in astrocytic endfeet (Figure S2C, blue asterisk). In both cohorts, we consistently observed that the short 3‐s whisker stimulus evoked slower astrocyte Ca^2+^ responses relative to the arteriole response. Arterioles dilated to their maximum approximately 1 to 2 s after stimulus onset, while all astrocyte Ca^2+^ releases required 4 to 4.5 s to reach their peak values (∼1 to 1.5 s after stimulus cessation).

A longer 30‐s whisker stimulus yielded more striking differences between AD and WT mice, particularly in astrocyte Ca^2+^ dynamics. Response profiles exhibited biphasic features for both arterial dilation and astrocyte Ca^2+^ release, including a rapid rising phase followed by a prolonged plateau phase. Consequently, we analyzed dilation and Ca^2+^ release both over the full stimulus duration (all 30 s) as well as the late phase (the final 2 s). Again, AD and WT mice experienced comparable peak diameter in arterioles within the first 3 s of stimulation. Following peak dilation, arterioles constricted slightly and plateaued to a smaller diameter (Figure 1J). For both phases, we observed no significant differences in arterial dilation kinetics, maximal diameter, or AUC between AD and WT mice (Figures 1J and S3A). Astrocyte Ca^2+^ responses differed more substantially between cohorts during the 30‐s stimulus (Figure 1K–M and Figure S3B–D). In astrocyte soma and endfeet, both AD and WT mice achieved similar peak values of Ca^2+^ release, but AD mice achieved their peak significantly earlier (Figure S3B–D). In WT mice, astrocytes persistently released Ca^2+^ from all compartments until stimulus cessation, while Ca^2+^ release in AD astrocyte peaked rapidly during the first ∼5 s of stimulation and diminished markedly thereafter. During the last 2 s of stimulation, somatic Ca^2+^ responses were significantly lower in AD mice compared to WT (Figure 1K). Endfeet and process Ca^2+^ responses also showed a reduction in AD mice, although these differences did not reach statistical significance (Figure 1 L,M, endfeet: *p* = 0.0796, process: *p *= 0.0678). Linear mixed‐effects modeling revealed faster Ca^2^^+^ dynamics in AD mice (Figure S3B–D, blue asterisk). The latency to peak Ca^2^^+^ response was significantly shorter across all three astrocytic compartments in AD mice. Rise time was also significantly reduced in the soma and processes (Figure S3B,D). In addition, somatic Ca^2^^+^ responses in AD mice exhibited shorter durations compared to WT (Figure S3B). Our observations indicate a compartmentalized influence of AD pathology to astrocyte Ca^2+^. In all microdomains, AD mice could not sustain astrocytic Ca^2+^ release in response to longer 30‐s sensory stimulation, suggesting an impaired capacity to sustain Ca^2^^+^ responses during prolonged stimulation.

**FIGURE 2:**
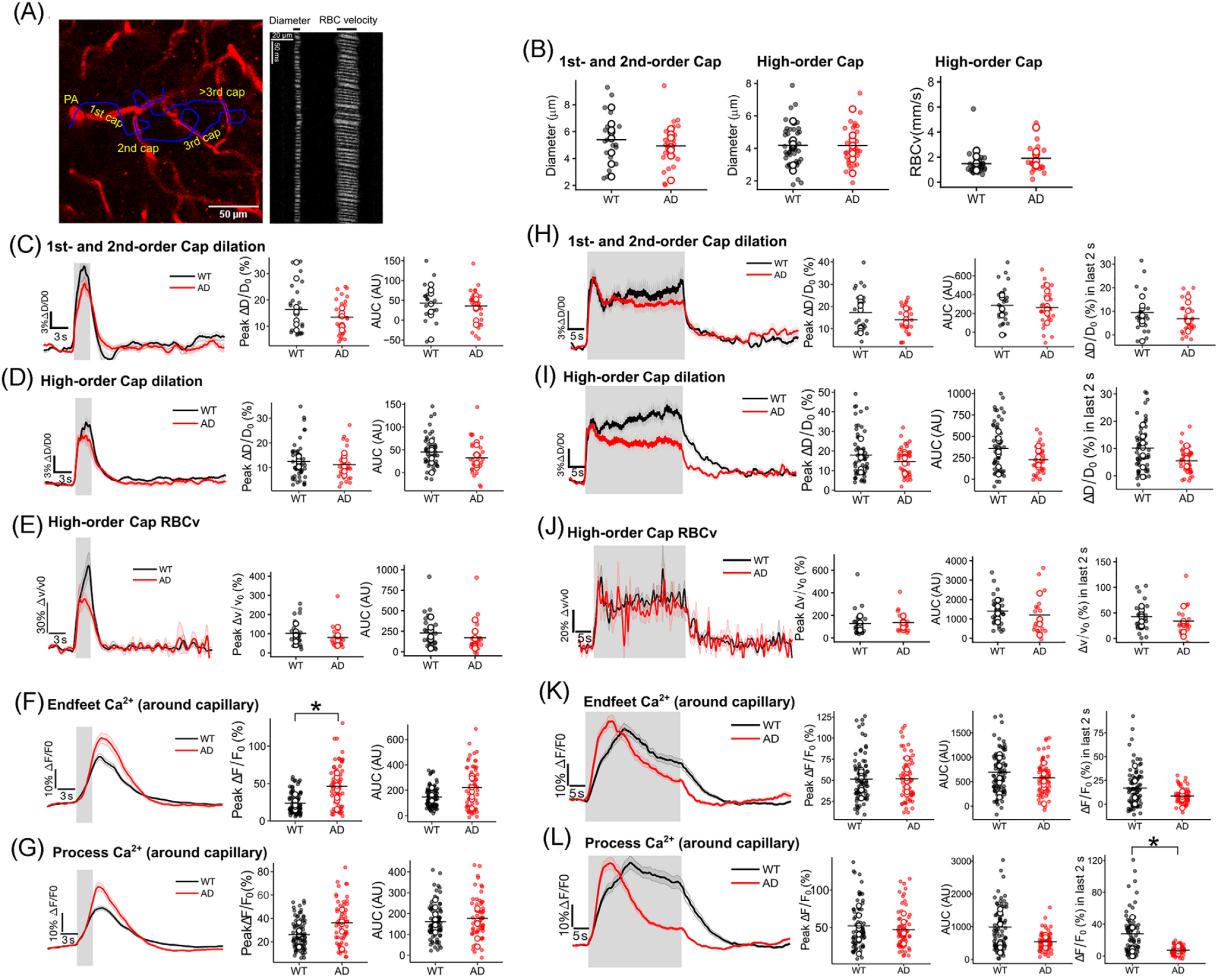
Baseline (pre‐LPS) capillary and astrocytic responses to sensory stimulation in the barrel cortex of AD and WT mice. (A) Two‐photon line‐scan measurements of capillary diameter and red blood cell (RBC) velocity. (B) Resting‐state capillary diameter and RBC velocity in lower‐order capillaries and high‐order capillaries under baseline (pre‐LPS) conditions. (C–F) Trial‐averaged time courses and quantification of capillary dilation (lower‐order and higher‐order capillaries), RBC velocity, and astrocyte Ca^2^^+^ responses (endfeet and processes) evoked by 3‐s whisker stimulation at baseline. (G–J) Trial‐averaged time courses and quantification of capillary dilation, RBC velocity, and astrocyte Ca^2^^+^ responses evoked by 30‐s whisker stimulation at baseline. Small dots represent individual ROIs, larger dots indicate per‐animal means, and horizontal lines denote the group mean across animals. Sample sizes are summarized in Tables S2 and S3.

The influence of astrocytes on microvascular blood flow reportedly extends beyond penetrating arterioles to laterally oriented pre‐capillary arterioles and capillaries.^45^, ^46^ In this dense network of small, more prevalent microvessels, even modest alterations to vessel diameter can substantially impact vascular resistance and total blood flow.^47^ To examine how amyloid pathology and associated hyperactive astrocytes affect hemodynamics in these vessels, we performed rapid two‐photon line scan measurements to measure capillary diameter and RBC speed (Figures 2A and S4). We detected strong vessel dilation in both lower‐order and higher‐order capillaries. Vessel dilation and RBC speed in laterally oriented arteriole branches and capillary branches extending from the downward penetrating arteriole (first order to fourth order) were measured and downsampled to a rate of 20 Hz. The vessels were bundled further: with first‐ to second‐order capillaries grouped as lower‐order capillaries and at third‐ or higher‐order grouped as higher‐order capillaries. Baseline vessel diameters and RBC speed were calculated as the mean values from 1.5 to 4.5 s within the 5 s baseline period (Figure 2B). AD mice showed comparable capillary diameter and RBC velocity compared to WT mice (Figure S5). For short (3 s) stimulation, no significant differences were observed in relative peak dilation or RBC velocity across capillary orders (Figure 2C–E). Rise time of high‐order capillary dilation in AD mice is significantly shorter than the WT mice (Figure S6B). Astrocytic endfeet surrounding capillaries exhibited higher peak Ca^2+^ responses (Figure 2F) and longer durations in AD mice (Figure S6D). During prolonged (30‐s) stimulation, no significant difference was found in capillary response between WT and AD (Figures 2H–J and Figure S7A,B). Nevertheless, higher‐order capillaries in AD mice showed a clearly reduced dilation, as indicated by the AUC and the dilation during the last 2 s of stimulation; however, the reduction was not significant as revealed by the linear mixed‐effects model (Figure 2I). Astrocytic Ca^2+^ responses exhibited distinct temporal alterations in AD mice (Figure 2K,L). In endfeet, peak Ca^2+^ responses were comparable between WT and AD mice, and no significant differences were observed in AUC or late‐phase responses (Figure 2K). In contrast, process Ca^2+^ signals in AD mice showed a markedly reduced sustained response, with significantly lower Ca^2+^ levels during the last 2 s of stimulation and a shorter duration (Figure 2L and Figure S7D), while peak amplitude and AUC remained comparable between groups (Figure 2L). Both Ca^2+^ release from endfeet and processes in AD mice have significantly shorter rise times compared to WT mice (Figure S7C,D). AD processes Ca^2+^ exhibited significantly shorter peak latency, indicating a faster kinetics in AD astrocytes (Figure S7D).

### 3.2 LPS‐induced inflammation alters spontaneous astrocytic Ca^2^^+^ and neurovascular coupling

To evaluate how prolonged systemic inflammation alters astrocyte Ca^2^^+^ signaling and its contributions to CBF regulation, animals underwent a 14‐day LPS‐induced inflammation challenge. We used two‐photon imaging to measure astrocyte Ca^2^^+^ dynamics and microvascular dilation under resting‐state conditions and during functional hyperemia in awake AD and WT mice. The same imaging FOVs were imaged on days 0, 7, and 14 of LPS administration.

**FIGURE 3:**
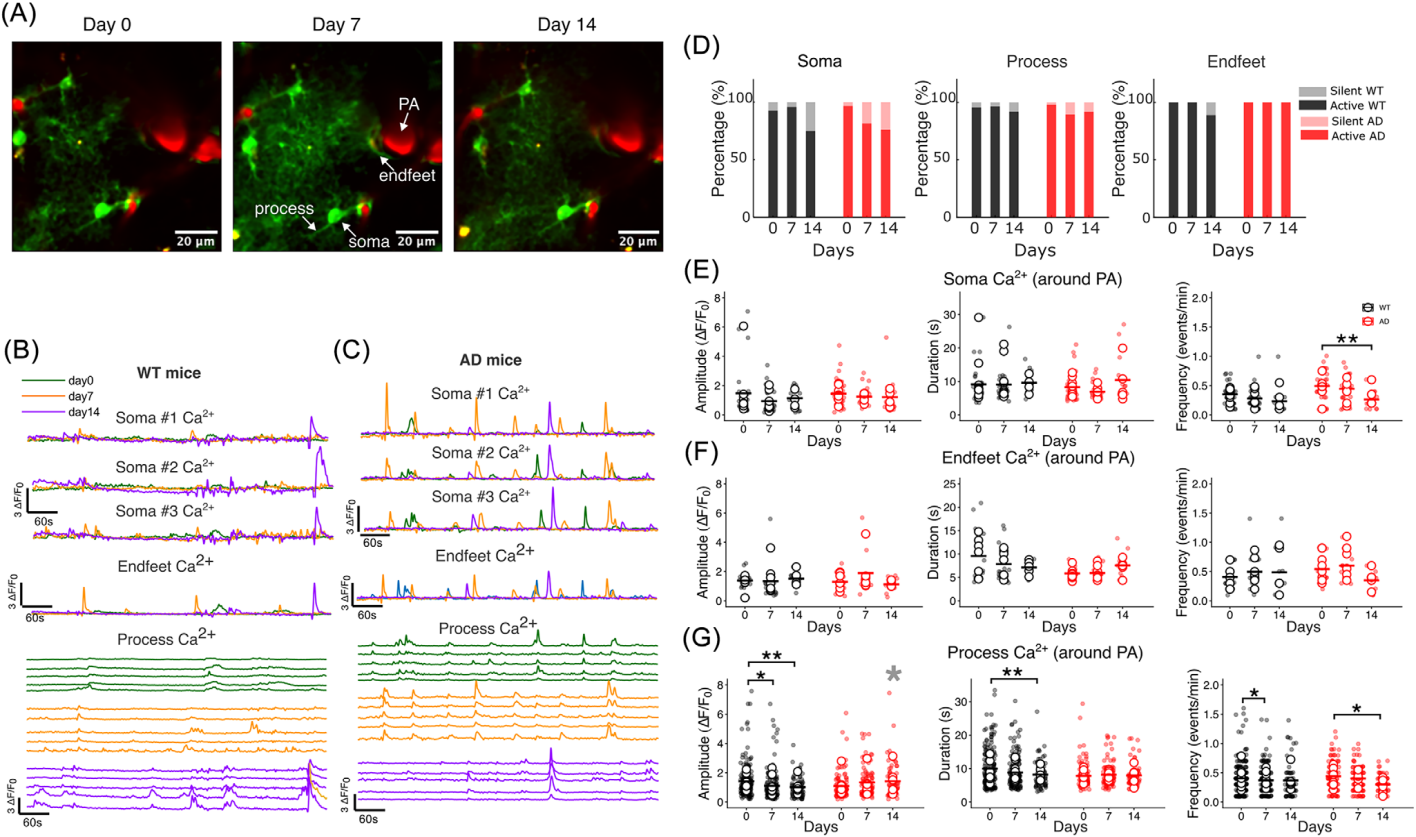
LPS‐induced inflammation alters spontaneous astrocyte Ca^2+^ activity in the cortex of AD and WT mice. (A) Average intensity projections of cortical vasculature and astrocytes at baseline (day 0) and after 7 and 14 days of LPS‐induced systemic inflammation in somatosensory cortex of an AD mouse. (B and C) Representative traces of spontaneous Ca^2+^ activity recorded from astrocyte compartments (soma, endfeet, and processes) in WT (B) and AD (C) mice at baseline (day 0) and following LPS treatment (days 7 and 14). (D) Proportion of active and silent astrocyte compartments across time points in WT and AD mice. (E–G) Quantification of spontaneous astrocyte Ca^2^^+^ activity, including amplitude, duration, and event frequency, across astrocyte compartments during LPS treatment. Small dots represent individual ROIs, larger dots indicate per‐animal means, and horizontal lines denote group mean across animals. Sample sizes are summarized in Table S1.

Figure 3A displays example measurements of resting‐state, spontaneous Ca^2^^+^ release from astrocytic compartments over the course of the 14‐day inflammatory threat. Raster scanning was performed in ∼88 µm^2^ cortical regions surrounding penetrating arterioles for 10 min at ∼4.4 Hz. We observed no discernible differences in astrocyte morphology from days 0 to 14. Dynamic Ca^2^^+^ transients were quantified and analyzed from the soma, processes, and endfeet compartments of astrocytes (Figure 3B,C). During the 14‐day period, we observed variations in spontaneous Ca^2^^+^ fluctuations in astrocytes in both cohorts, with some cell compartments demonstrating no Ca^2^^+^ transients over the 10‐min measurement. Figure 3D illustrates how the proportion of these silent, quiescent cell compartments changed during the inflammatory period. Among the cells that remained active in both AD and WT cohorts, the frequency of spontaneous Ca^2^^+^ spikes progressively decreased during the 14‐day period within astrocytic soma and process compartments (Figure 3E,G). WT mice also showed significantly reduced amplitude and duration of process Ca^2^^+^ relsease (Figure 3G). No substantial changes were observed in endfeet Ca^2^^+^ (Figure 3F).

#### 3.2.1 Penetrating arterioles and neighboring astrocytes

To determine whether prolonged systemic inflammation impacts astrocytic contributions to functional hyperemia, we examined dilation of penetrating arterioles and astrocyte Ca^2^^+^ release in response to brief (3‐s) and sustained (30‐s) pneumatic whisker stimulation.

**FIGURE 4:**
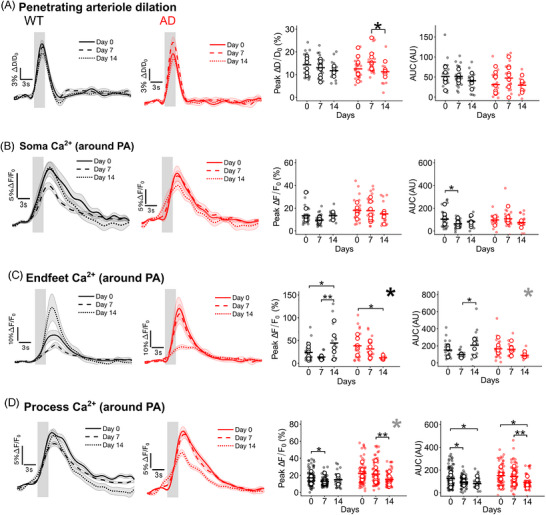
LPS‐induced inflammation alters brief sensory‐evoked penetrating arteriole dilation and astrocyte Ca^2^^+^ responses. (A) Trial‐averaged time courses and quantification of 3‐s whisker stimulation evoked penetrating arteriole dilation in WT and AD mice at baseline (day 0) and after 7 and 14 days of LPS treatment. (B–D) Trial‐averaged time courses and quantification of compartment‐specific astrocyte Ca^2^^+^ responses (soma, endfeet, and processes) evoked by 3‐s whisker stimulation in WT and AD mice across time points (days 0, 7, and 14). Small dots represent individual ROIs, larger dots indicate per‐animal means, and horizontal lines denote the group mean across animals. Statistical analyses were performed using a linear mixed‐effects model. Large asterisk (★) indicates a significant genotype × day interaction from linear mixed‐effects modeling (black: *p* < 0.001, gray: *p* < 0.01). Sample sizes are summarized in Table S4.

For the 3‐s stimulus, arteriole dilation responses were largely preserved across the 14‐day LPS period in both AD and WT mice, with no significant differences between days 0 and 14 in either genotype. In AD mice, however, peak dilation was significantly reduced between days 7 and 14 (Figure 4A), suggesting that prolonged inflammatory exposure selectively attenuates brief‐stimulus arteriole responses at later time points in the AD brain.

In contrast, astrocytic Ca^2^^+^ responses to brief 3‐s whisker stimulation showed more pronounced and compartment‐specific alterations. In astrocytic soma, WT mice showed a significant reduction in AUC between days 0 and 7, while AD mice showed no significant changes in somatic Ca^2^^+^ amplitude or kinetics over the LPS period (Figures 4B and S2B).

Within astrocytic endfeet, LPS induced opposing amplitude changes in the two genotypes: WT mice showed a significant increase in peak Ca^2^^+^, while AD mice showed a significant reduction (Figure 4C). Linear mixed‐effects modeling confirmed significant genotype × day interactions for both peak ΔF/F_0_ and AUC, indicating that WT and AD endfeet followed divergent trajectories under inflammatory challenge. Endfeet kinetics were also significantly altered: Peak latency increased significantly in both genotypes, while response duration and rise time showed significant genotype × day interactions, with rise time being significantly prolonged specifically in AD mice (Figure S2C).

In astrocyte processes, systemic inflammation provoked significant reductions in peak Ca^2^^+^ release and AUC in both AD and WT mice (Figure 4D). A significant genotype × day interaction was observed for peak ΔF/F_0_, indicating that the temporal trajectory of process Ca^2^^+^ amplitude differed between genotypes over the course of the inflammatory threat. Rise time was significantly altered in both genotypes and showed a significant genotype × day interaction. Peak latency did not reach significance within either genotype individually; however, a significant genotype × day interaction was detected, indicating that LPS differentially reshapes the temporal profile of process Ca^2^^+^ responses in AD and WT mice (Figure S2D). Together, these compartment‐specific findings demonstrate that systemic inflammation disrupts astrocyte Ca^2^^+^ signaling in a genotype‐dependent manner, with particularly divergent effects observed in endfeet and processes.

**FIGURE 5:**
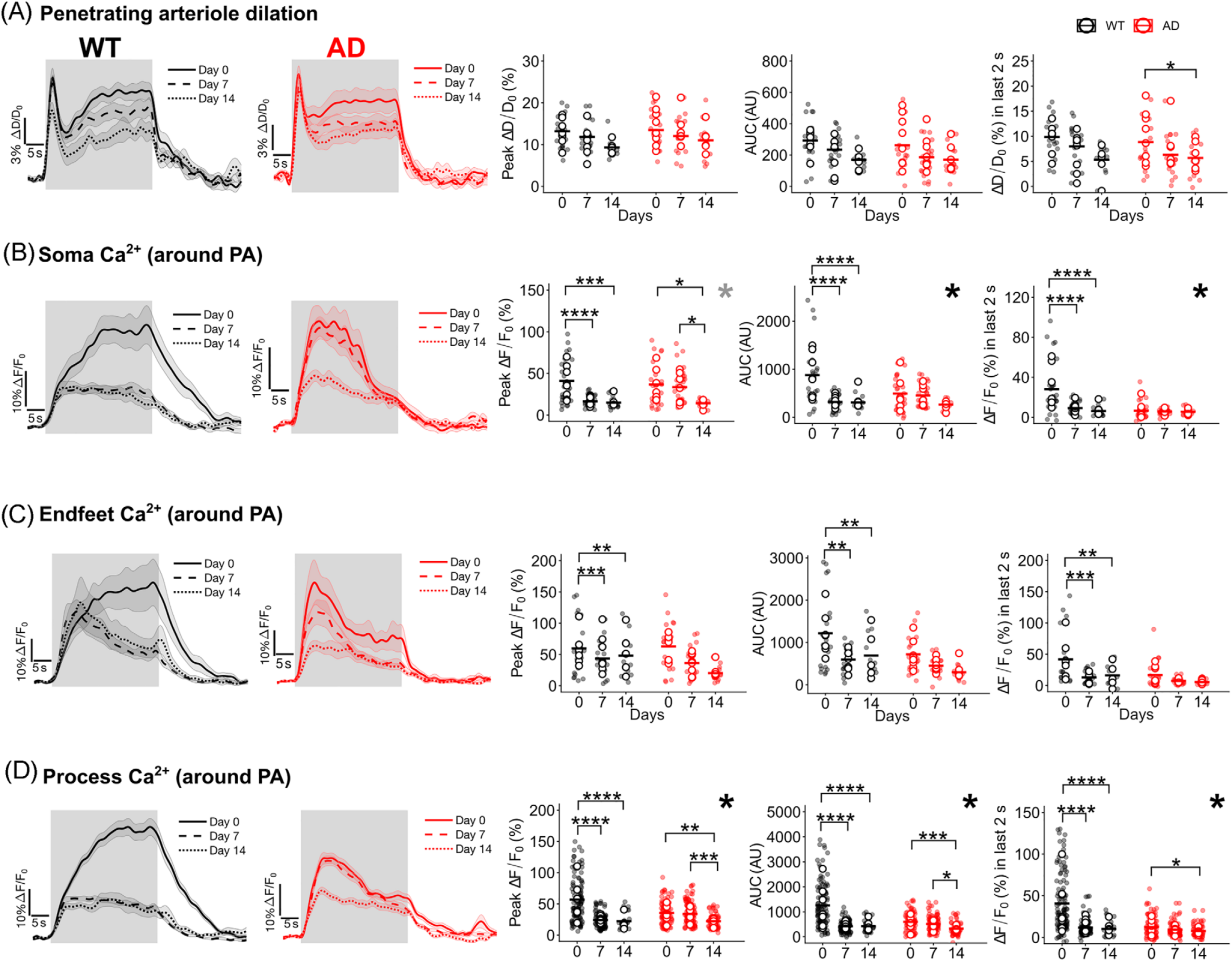
LPS‐induced inflammation alters prolonged sensory‐evoked penetrating arteriole dilation and astrocyte Ca^2^^+^ responses. (A) Trial‐averaged time courses and quantification of 30‐s whisker stimulation evoked penetrating arteriole dilation in WT and AD mice at baseline (day 0) and after 7 and 14 days of LPS treatment. (B–D) Trial‐averaged time courses and quantification of compartment‐specific astrocyte Ca^2^^+^ responses (soma, endfeet, and processes) evoked by 30‐s whisker stimulation in WT and AD mice across time points (days 0, 7, and 14). Small dots represent individual ROIs, larger dots indicate per‐animal means, and horizontal lines denote the group mean across animals. Statistical analyses were performed using a linear mixed‐effects model. Large asterisk (★) indicates a significant genotype × day interaction from linear mixed‐effects modeling (black: *p* < 0.001). Sample sizes are summarized in Table S5.

In response to the prolonged 30‐s stimulus, arteriole dilation amplitude was largely preserved across the LPS period in both genotypes, with no significant changes in peak dilation or AUC in either WT or AD mice. However, late‐phase dilation during the final 2 s of stimulation was significantly reduced in AD mice between days 0 and 14 (Figure 5A), indicating progressive vulnerability of sustained arteriole responses specifically in the AD brain. In WT mice, arteriole rise time was significantly shortened between days 0 and 14 and between days 7 and 14 (Figure S3A), suggesting LPS‐induced changes in arteriole dilation kinetics in WT that are distinct from the amplitude changes observed in AD.

Around penetrating arterioles, astrocytic Ca^2^^+^ responses to 30‐s stimuli showed more pronounced and widespread LPS‐induced changes compared to vascular responses, with compartment‐specific patterns. In astrocytic soma, WT mice showed significant progressive reductions in peak ΔF/F_0_, AUC, and late‐phase Ca^2^^+^ release, with significant differences between days 0 and 7 and days 0 and 14. AD mice showed significant reductions in peak ΔF/F_0_ between days 0 and 14 and days 7 and 14, indicating a delayed but progressive suppression relative to WT. Significant genotype × day interactions were detected for peak ΔF/F_0_, AUC, and late‐phase Ca^2^^+^ release, confirming divergent temporal trajectories between genotypes (Figure 5B). Somatic Ca^2^^+^ kinetics were also significantly altered: Peak latency was significantly reduced in WT mice between days 0 and 7 and days 0 and 14, and WT and AD mice differed significantly at the onset of the LPS period (day 0). Response duration also differed significantly between genotypes at day 0. Rise time was significantly shortened in WT mice between days 0 and 7 and days 0 and 14, and WT and AD mice differed significantly at day 0, with a significant genotype × day interaction (Figure S3B).

LPS provoked significant reductions to astrocytic endfoot responses to 30‐s whisker stimulation, including peak ΔF/F_0_, AUC, and late‐phase Ca^2^^+^ release in WT mice between days 0 and 7 and days 0 and 14. In AD mice, however, no changes in the amplitude of astrocytic endfoot response were detected over the course of 14 days of inflammatory threat (Figure 5C). Peak latency was significantly reduced in WT mice between days 0 and 7, and WT and AD mice differed significantly at day 0. Rise time was significantly shortened in WT mice between days 0 and 7 (Figure S3C).

In astrocyte processes, both WT and AD mice showed significant LPS‐induced reductions across multiple amplitude metrics for 30‐s stimulation. WT mice showed significant reductions in peak ΔF/F_0_, AUC, and late‐phase Ca^2^^+^ release between days 0 and 7 and days 0 and 14. AD mice showed significant reductions in peak ΔF/F_0_ between days 0 and 14 and days 7 and 14, in AUC between days 0 and 14 and days 7 and 14, and in late‐phase Ca^2^^+^ release between days 0 and 14. Significant genotype × day interactions were detected for peak ΔF/F_0_, AUC, and late‐phase Ca^2^^+^ release (Figure 5D). Process kinetics were also significantly affected: Peak latency was significantly reduced in WT mice between days 0 and 7 and days 0 and 14, and in AD mice between days 0 and 7 and days 0 and 14, with WT and AD mice also differing significantly at the onset of the LPS period. Response duration was significantly reduced in AD mice between days 0 and 7. Rise time was significantly shortened in WT mice between days 0 and 7 and days 0 and 14, and WT and AD mice differed significantly on day 0 (Figure S3D).

#### 3.2.2 Capillaries and neighboring astrocytes

We also examined how LPS‐induced inflammation affects capillary hemodynamics and surrounding astrocyte Ca^2^^+^ responses during resting state and during brief and prolonged functional hyperemia. Resting‐state capillary diameter and RBC velocity did not change significantly across days 0, 7, and 14 in either WT or AD mice for either capillary order (Figure S5), indicating that the 14‐day LPS paradigm did not induce overt changes in resting capillary structure or hemodynamics.

**FIGURE 6:**
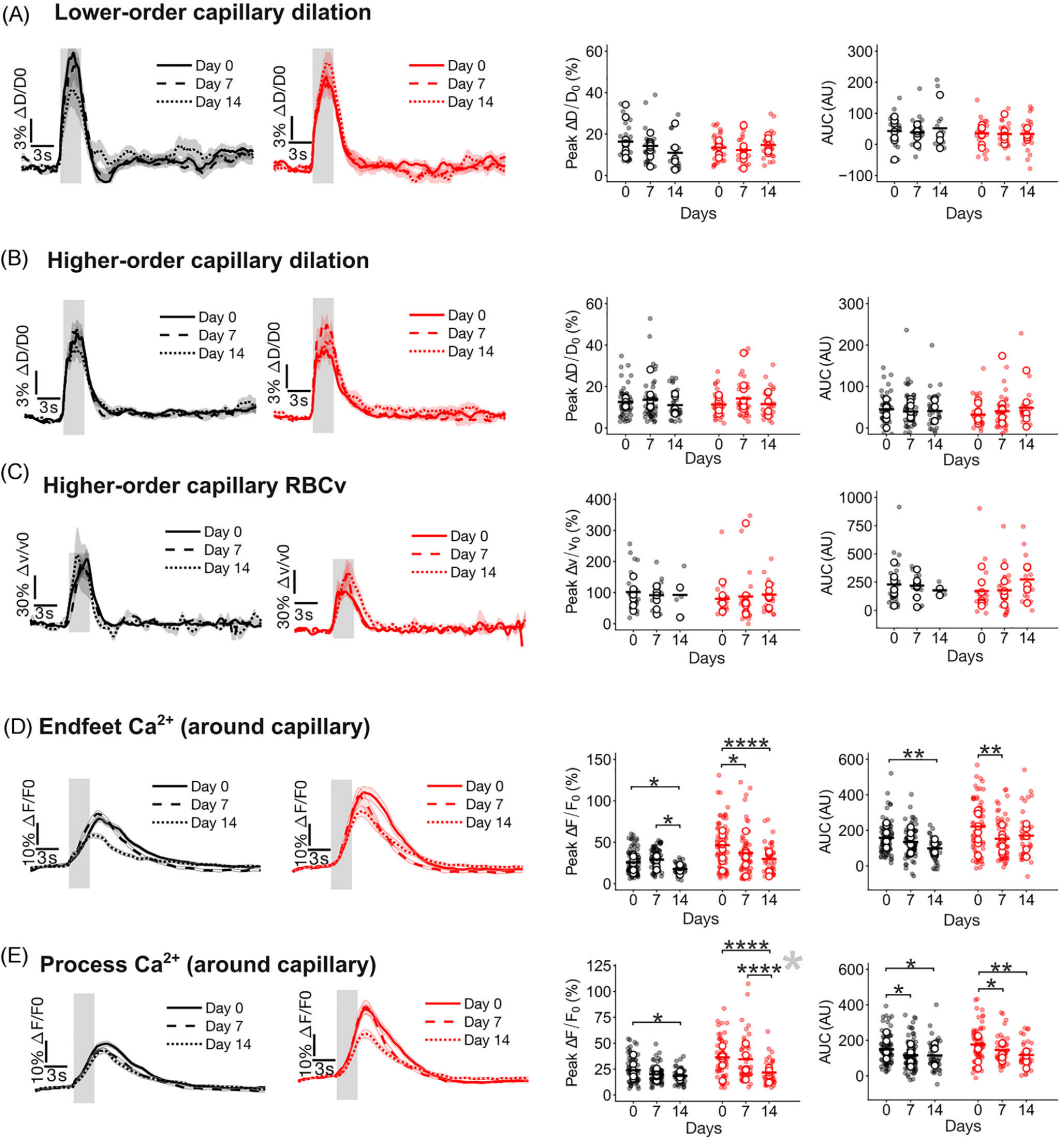
LPS‐induced inflammation alters capillary and astrocytic responses to brief whisker stimulation. (A–C) Two‐photon line‐scan measurement of 30‐s whisker stimulation‐evoked capillary responses. (A) Dilation (ΔD/D_0_,%) in lower‐order capillary. (B) Dilation in higher‐order capillary (≥3rd order). (C) Red blood cell (RBC) velocity changes (Δv/v, %) in higher‐order capillaries. Peak amplitude and the area under curve of the responsive traces were quantified. (D and E) Trial‐averaged time courses and quantification of astrocyte Ca^2^^+^ responses in endfeet (D) and processes (E), measured from regions of interest (ROIs) surrounding capillaries during whisker stimulation. Small dots represent individual ROIs, larger dots indicate per‐animal means, and horizontal lines denote the group mean across animals. Large asterisk (★) indicates a significant genotype × day interaction from linear mixed‐effects modeling (light gray: *p* < 0.05). Statistical analyses were performed using a linear mixed‐effects model. Sample sizes are summarized in Table S2.

During brief (3‐s) whisker stimulation, stimulus‐evoked capillary dilation amplitude and RBC velocity did not show significant changes across the LPS period in either genotype for lower‐order or higher‐order capillaries (Figure 6A–C). However, in higher‐order capillaries, both the overall RBC velocity response and its rise time showed significant genotype × day interactions, indicating that WT and AD capillaries follow divergent temporal trajectories in their microvascular velocity responses over the course of LPS administration, despite preserved response amplitudes (Figure S6C). Additionally, higher‐order capillary dilation rise time was significantly shorter in AD mice compared to WT at the onset of the LPS period, suggesting that capillary dilation kinetics differ between genotypes under early inflammatory conditions (Figure S6B).

Astrocytic Ca^2^^+^ responses measured from endfeet and processes surrounding capillaries showed more consistent LPS‐induced changes during the 3‐s stimulus. Both endfeet and process peak ΔF/F_0_ and AUC were significantly reduced by LPS in both WT and AD mice (Figure 6D,E), indicating that systemic inflammation suppresses stimulus‐evoked Ca^2^^+^ release at the capillary level across genotypes. Process Ca^2^^+^ additionally showed a significant genotype × day interaction for peak ΔF/F_0_, reflecting divergent temporal trajectories between WT and AD mice over the course of LPS administration (Figure 6E). Regarding Ca^2^^+^ kinetics near capillaries, endfeet Ca^2^^+^ response duration was significantly shorter in AD mice compared to WT at the onset of the LPS period, while endfeet peak latency increased significantly in WT mice between days 0 and 7 (Figure S6D). Process rise time was significantly reduced in WT mice over the LPS period (Figure S6E). Together, these findings indicate that while capillary hemodynamic amplitudes are largely preserved during brief stimulation, astrocytic Ca^2^^+^ signaling at the capillary level is consistently suppressed in both genotypes under LPS, with genotype‐dependent differences emerging in the temporal dynamics of both microvascular velocity responses and process Ca^2^^+^ amplitude trajectories.

**FIGURE 7:**
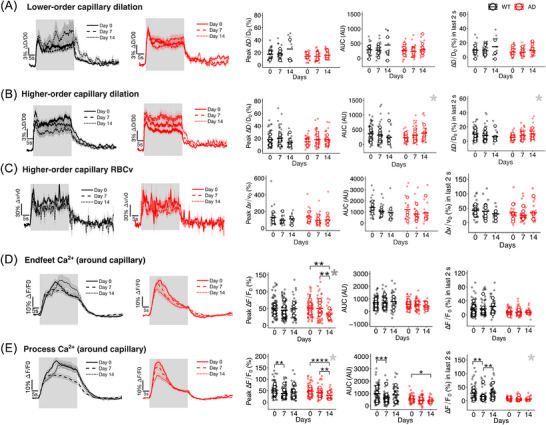
LPS‐induced inflammation alters capillary and astrocytic responses to prolonged whisker stimulation. (A–C) Two‐photon line‐scan measurement of 30‐s whisker stimulation‐evoked capillary responses. (A): Dilation (ΔD/D,%) in lower‐order capillary. (B) Dilation in higher‐order capillary ((≥3^rd^ order), and (C) Red blood cell (RBC) velocity changes (Δv/v,%) in higher‐order capillaries. Peak amplitude and the area under curve of the responsive traces were quantified. (D–E) Trial‐averaged time courses and quantification of astrocyte Ca^2^^+^ responses in endfeet (D) and processes (E), measured from regions of interest (ROIs) surrounding capillaries during whisker stimulation. Small dots represent individual ROIs, larger dots indicate per‐animal means, and horizontal lines denote the group mean across animals. Statistical analyses were performed using a linear mixed‐effects model. Sample sizes are summarized in Table 2. Large asterisk (★) indicates a significant genotype × day interaction from linear mixed‐effects modeling (light gray: *p* < 0.05). Black asterisks indicate significant pairwise comparisons from post hoc Tukey tests (*p < 0.05, **p < 0.01, ***p < 0.001, ****p < 0.0001).

Under prolonged 30‐s stimulation, higher‐order capillary dilation showed significant genotype × day interactions for both AUC and late‐phase dilation amplitude, reflecting divergent trajectories between WT and AD mice over the LPS period, with WT mice showing a modest declining trend and AD mice showing a modest increasing trend (Figure 7B). Higher‐order capillary dilation peak latency was significantly decreased in WT mice between days 0 and 7 (Figure S7B). RBC velocity in higher‐order capillaries remained unaffected under 14 days of LPS treatment (Figure 7C).

Astrocytic Ca^2^^+^ responses surrounding capillaries were substantially altered by LPS during prolonged stimulation. In endfeet, peak ΔF/F_0_ was significantly reduced in AD mice between days 0 and 14 and days 7 and 14, with a significant genotype × day interaction (Figure 7D). Endfeet Ca^2^^+^ kinetics also showed significant genotype × day interactions for both duration and rise time, with WT and AD mice differing significantly in rise time at the onset of the LPS period (Figure S7C).

In processes surrounding capillaries, peak ΔF/F_0_ was significantly reduced in WT mice between days 0 and 7 and in AD mice between days 0 and 14 and days 7 and 14, with a significant genotype × day interaction. AUC was significantly reduced in WT mice between days 0 and 7 and in AD mice between days 0 and 14. Late‐phase Ca^2^^+^ release was significantly reduced in WT mice between days 0 and 7 and days 7 and 14 but was not affected in AD mice, resulting in a significant genotype × day interaction (Figure 7E). Process Ca^2^^+^ kinetics showed significant genotype × day interactions for rise time, with WT and AD mice differing significantly in peak latency, duration, and rise time at the onset of the LPS period. Response duration was significantly reduced in AD mice between days 0 and 14 and days 7 and 14 (Figure S7D).

Together, these findings demonstrate that prolonged systemic inflammation preferentially disrupts astrocytic Ca^2^^+^ signaling relative to vascular responses at both the arteriole and capillary levels, with genotype‐dependent differences in the magnitude, timing, and trajectory of these effects. The results are consistent with prior work demonstrating that astrocytic Ca^2^^+^ contributes primarily to the modulation and maintenance of sustained functional hyperemia rather than its initiation.^38^

### 3.3 Ex vivo immunofluorescence analysis of LPS‐induced inflammation and $A\beta_{1-42}$ accumulation

To provide a preliminary histological assessment of neuroinflammatory and amyloid pathology changes following LPS administration, we harvested brains from a subset of mouse cohorts for IF staining of GFAP, Iba‐1, and Aβ_1‐42_ (*n* = 3 per cohort). We also analyzed a sham group of mice that received 14 days of saline injections (*n* = 3 for AD, *n* = 2 for WT) and an additional group of mice that received 7 days of LPS injection (*n* = 2 for AD, *n* = 3 for WT). Importantly, these analyses were designed to serve as exploratory, complementary observations rather than a comprehensive assessment. The mice that received 7 days of LPS injection were not used for in vivo imaging. Figure S8A displays representative confocal images of IF staining. We quantified GFAP expression in astrocytes and Iba‐1 expression in microglia by calculating the percentage area of GFAP‐ and Iba‐1‐expressed cells in the confocal images. In the saline control group, AD mice showed significantly higher GFAP expression in both cortex and hippocampus compared to WT mice, indicating a pre‐existing neuroinflammatory state in AD mice (Figure S8B). Iba‐1 expression was slightly, but not significantly, higher in the AD control group compared to WT control mice. After 7 days of LPS injection, GFAP and Iba‐1 expression in the WT brain both increased, with a statistically significant increase observed in GFAP expression. Interestingly, GFAP expression in AD astrocytes decreased slightly after 7 days of LPS injection, while microglial Iba1 expression increased after 7 days of LPS injection. Iba‐1 expression returned to baseline on day 14 in both cohorts. This pattern of partial recovery is consistent with our prior observations in younger APP/PS1dE9 mice at 8 months of age, in which cerebral capillary oxygenation and oxygen extraction showed a similar tendency toward recovery at day 14 of LPS administration, despite pronounced inflammatory disruptions at day 7.^27^ The similarities of these findings across age groups suggest that the temporal dynamics of immune tolerance induction are preserved across disease stages in this model and that the partial resolution of inflammatory markers at later time points reflects a consistent biological phenomenon rather than an age‐specific response.^48^, ^49^, ^50^ Figure S8C shows the representative wide‐field fluorescence imaging of Aβ_1‐42_ in the cortex and hippocampus of AD mice. Strikingly, 14 days of LPS injection yielded reduced $A\beta_{1-42}$ load in AD mouse brain, showing statistical significance in cortex and a strong trend in the hippocampus (Figure S8D). LPS injection induced $A\beta_{1-42}$ reduction was also reported by other studies.^51^, ^52^, ^53^ Higher $A\beta_{1-42}$ load was observed in the cortex compared to hippocampus (Figure S8D), agreeing well with previous reports in APPswe/PS1dE9 mice.^54^, ^55^

## 4 DISCUSSION

Our findings show that amyloid pathology is associated with attenuated astrocytic Ca^2^^+^ signaling during sensory stimulation – particularly during sustained stimulation – alongside largely preserved stimulus‐evoked vessel dilation. Under secondary inflammatory challenge, LPS further disrupted both baseline and stimulus‐evoked astrocytic Ca^2^^+^ responses across compartments, whereas vascular hemodynamic responses were comparatively less affected, with significant effects confined to late‐phase arteriole dilation and capillary dilation trajectories during prolonged stimulation. Linear mixed‐effects modeling revealed significant genotype × day interactions, indicating that AD and WT mice followed divergent temporal trajectories in their responses to sustained inflammatory challenge.

### 4.1 Astrocytic Ca^2^^+^, microvascular blood flow, and AD

Longstanding debates continue regarding the precise contributions of astrocyte Ca^2^^+^ to resting‐state CBF and functional hyperemia. Under resting conditions, astrocytic Ca^2^^+^ helps regulate arteriole diameter, and during focal stimulation, glutamate‐triggered Ca^2^^+^ increases stimulate release of vasoactive compounds that modulate arteriole and capillary diameters.^6^, ^44^, ^56^, ^57^, ^58^, ^59^ However, astrocyte Ca^2^^+^ has also been shown to induce both dilation and constriction depending on metabolic context.^60^ Reports indicate that somatic Ca^2^^+^ release is too slow to initiate arteriole dilation,^61^, ^62^ while faster endfeet and process transients play more direct roles in vascular regulation.^45^, ^46^, ^63^, ^64^, ^65^, ^66^ Our findings in 12‐ to 13‐month‐old awake female mice support the emerging hypothesis that astrocytes primarily contribute to the modulation and maintenance of sustained hemodynamic response, rather than its initiation.^40^

Previous studies of AD mouse models reported both hyperactive and diminished astrocytic Ca^2+^ signaling, largely dependent on brain region, disease stage, the presence or absence of functional stimulation, and animal model.^13^, ^67^, ^68^, ^69^, ^70^ In the absence of systemic inflammation, our observations indicate slight alterations in APP/PS1 mice that were compartment‐ and stimulus‐dependent. Stimulus‐evoked cerebral blood volume changes and penetrating arteriole dilation were largely comparable between AD and WT mice, consistent with preserved neurovascular coupling reported in other amyloid models at similar ages.^71^, ^72^ Although capillary dilation and RBC velocity were largely preserved, higher‐order capillaries demonstrated significantly shorter rise times and trends toward reduced dilation and faster relaxation during prolonged stimulation. The results support emerging evidence that vascular impairment in AD may originate in the microcirculation before propagating to larger vessels.^73^, ^74^, ^75^ In APP/PS1 mice, cerebral amyloid angiopathy (CAA) has been observed as early as 6 months, causing increased vessel stiffness, vascular smooth muscle cell loss, and basement membrane disruption. While CAA is believed to reduce reactivity in pial arterioles, we observed largely preserved responses from penetrating arterioles. This is consistent with evidence that CAA‐associated pathological changes are more pronounced in surface vessels and may require more advanced disease stages to substantially compromise penetrating arteriole reactivity.^74^, ^75^

### 4.2 Systemic inflammation's impact on neurovascular coupling and astrocytic Ca^2^^+^ signaling

In healthy animals, acute LPS‐induced inflammation (4 to 24 h) yields substantial reductions in capillary blood flow, cerebral blood volume, and CMRO_2_ responses during functional hyperemia.^24^, ^76^ Here, 14 days of low‐dose LPS resulted in modest hemodynamic alterations overall, with significant effects confined to specific metrics: Peak arteriole dilation declined between days 7 and 14 in AD mice during brief stimulation, late‐phase arteriole dilation was significantly reduced in AD mice on day 14 during prolonged stimulation, and significant genotype × day interactions emerged in higher‐order capillary AUC and late‐phase dilation during prolonged stimulation – reflecting divergent rather than uniformly impaired trajectories. The milder overall vascular effects relative to prior reports are consistent with the lower LPS dose used here (0.3 mg/kg vs >1 mg/kg).

Our findings indicate that prolonged inflammation diminished astrocyte Ca^2^^+^ dynamics more substantially than microvascular hemodynamics. To our knowledge, neither acute nor chronic systemic inflammation's influence on astrocytic Ca^2^^+^ signaling was previously investigated in vivo. In both AD and WT mice, we observed persistent, compartment‐specific reductions in Ca^2^^+^ release under resting‐state conditions and during functional stimulation, with LPS‐induced suppression more pronounced during 30‐s than 3‐s stimulation, indicating that secondary inflammatory challenge preferentially impairs astrocytes' capacity to sustain prolonged evoked Ca^2^^+^ responses.

Our observations are consistent with the emerging hypothesis that AD pathology increases astrocytes’ susceptibility to a secondary inflammatory challenge^77^ and highlight the potential impact of recurrent systemic inflammatory insults on neurovascular function during preclinical AD progression. The results strongly motivate mechanistic studies to directly assess how inflammatory challenges alter the molecular mediators of astrocytic Ca^2+^ homeostasis and release, along with potential approaches to restoring their function. Among the most relevant candidates are G protein‐coupled receptors and their downstream effectors, which mediate ATP‐ and glutamate‐evoked Ca^2^^+^ responses and are frequently dysregulated under neuroinflammatory conditions.^78^, ^79^ Impairment of ER Ca^2^^+^ homeostasis represents another plausible mechanism. STIM1, which gates Orai1‐mediated store‐operated Ca^2^^+^ entry, is downregulated in AD models, and its rescue restores astrocytic Ca^2^^+^ dynamics and synaptic plasticity,^67^ warranting further investigation of inflammatory insults on STIM1‐dependent store refilling. Orai1 depletion reduces LPS‐induced astrocytic pro‐inflammatory cytokine release and Ca^2^^+^ oscillation frequency,^80^ and future studies employing astrocyte‐specific Orai1 manipulation or CalEx‐mediated Ca^2^^+^ suppression will help establish direct causal links between observed astrocytic Ca^2^^+^ and the vascular phenotypes observed here.

The inflammaging phenomenon – in which age‐related increases in pro‐inflammatory mediators gradually lower the threshold for neuroinflammatory responses – provides context for understanding why AD and WT mice responded differently to secondary inflammatory challenge.^22^ Our observed divergent trajectories reflect distinct neuroimmune phenotypes: WT mice showed earlier and more pronounced Ca^2^^+^ suppression with significant changes by day 7, while AD mice exhibited a delayed pattern with significant suppression emerging primarily by day 14. For vascular responses, late‐phase arteriole dilation was selectively vulnerable in AD mice but not WT, indicating that cumulative vascular effects emerge later and more persistently in the AD brain. One interpretation is that chronic amyloid pathology attenuates AD astrocytes' capacity to respond dynamically to a new inflammatory stimulus – a ceiling effect consistent with prior reports of attenuated microglial LPS responses in aged APP/PS1 mice.^81^ Importantly, reduced early response magnitude in AD does not indicate reduced vulnerability; it reflects a distinct inflammatory phenotype shaped by pre‐existing pathology.

Consistent with previous reports,^49^, ^51^ our pilot immunofluorescence observations indicate reduced Aβ levels following repeated LPS exposure in APP/PS1 mice, suggesting that chronic low‐dose systemic inflammation may enhance immune‐mediated Aβ clearance, most likely through microglial activation rather than astrocytic Ca^2^^+^ signaling directly. This reduction occurred alongside impaired astrocyte physiology, highlighting a dissociation between amyloid burden and astrocyte‐mediated physiological support. Systemic inflammation has also been shown to disrupt BBB integrity and reduce LRP1‐mediated Aβ efflux,^82^ though the net effect on Aβ dynamics depends on dose and duration – under the chronic low‐dose paradigm used here, enhanced microglial clearance likely outweighed any transport‐mediated Aβ accumulation.

The limitations of this study warrant consideration. Neuronal activity was not directly measured; enhanced neuronal drive in APP/PS1 mice or under LPS could contribute to preserved hyperemic responses despite reduced astrocytic Ca^2^^+^ signaling, and future studies incorporating simultaneous neuronal recordings will be needed to clarify these interactions.^83^, ^84^ Astrocyte selection based on vessel proximity rather than plaque proximity introduces spatial heterogeneity that may increase Ca^2^^+^ measurement variance, though group‐level analyses consistently revealed significant alterations in APP/PS1 mice. The ∼6% relative dilation responses in higher‐order capillaries correspond to absolute diameter changes of approximately 0.3 to 0.5 µm, approaching the optical resolution limit of two‐photon microscopy. Group‐averaged responses across multiple trials and animals mitigate but do not eliminate this constraint, and linear mixed‐effects modeling did not reveal statistically significant dilation amplitudes in higher‐order capillaries. GCaMP6f expression via the GfaABC1D promoter may theoretically be influenced by LPS‐induced reactive astrogliosis, though per‐ROI ΔF/F_0_ normalization and adaptive thresholding introduce a conservative rather than inflationary bias. Vascular density and morphological remodeling were not directly quantified, and inflammatory exposure may reduce microvascular density and perivascular coverage in ways that could independently influence neurovascular responses.^85^ The IF analysis was intentionally limited in scope and animal numbers. Finally, this study was conducted exclusively in female mice; sex differences in astrocytic Ca^2^^+^ responses to amyloid pathology and systemic inflammation remain incompletely characterized and represent an important direction for future investigation.

The present findings motivate several directions for future work. A systematic comparison across disease stages – including earlier time points where amyloid pathology is still evolving and astrocyte reactivity may not yet be saturated – would clarify whether the Ca^2^^+^ dysregulation and neurovascular vulnerabilities observed here emerge progressively or plateau at advanced stages and could identify windows where intervention may be most tractable. Direct causal interrogation of the astrocyte Ca^2^^+^–vascular relationship should be pursued through selective manipulation approaches: CalEx‐mediated Ca^2^^+^ suppression would isolate the contribution of astrocytic Ca^2^^+^ to the sustained hyperemic response under inflammatory conditions, while STIM1 overexpression or Orai1 modulation could test whether restoring intracellular Ca^2^^+^ store dynamics rescues neurovascular function in the context of combined amyloid pathology and systemic inflammation. Simultaneous two‐photon Ca^2^^+^ imaging and electrophysiological or GCaMP‐based neuronal recording would allow direct dissociation of neuronal and astrocytic contributions to preserved and impaired hemodynamic responses. Finally, extending this experimental paradigm to male mice and to alternative inflammatory models beyond LPS would broaden the generalizability of these findings and delineate sex‐ and context‐dependent dimensions of astrocyte‐mediated neurovascular dysfunction in preclinical AD.

## CONFLICT OF INTEREST STATEMENT

The authors declare no competing interests. Author disclosures are available in the Supporting Information.

## CONSENT STATEMENT

Consent was not necessary for this study.

## Supporting information




**Supporting information**: alz71607‐supp‐0001‐SuppMat.docx

**Supporting information**: alz71607‐supp‐0002‐SuppMat.pdf
