# Supplementary material for: Prolonged systemic inflammation worsens impairments to astrocyte Ca2+ and functional hyperemia in Alzheimer's disease
Source: Alzheimers Dement. 2026 Jul 23;22(7):e71607. doi: 10.1002/alz.71607 (PMC13393075; doi:10.1002/alz.71607)
Supplement: Supplementary file 1 — Supporting information: alz71607‐supp‐0001‐SuppMat.docx [file ALZ-22-e71607-s001.docx]

**Supplemental information**

**Prolonged systemic inflammation worsens impairments to astrocyte Ca^2+^ and functional hyperemia in Alzheimer’s disease**

**Chang Liu, Kimia Sakha, Jaime Anton, Alfredo Cardenas-Rivera, Mohammad A. Yaseen**

**Department of Bioengineering, Northeastern University, Boston, MA 02115, USA**


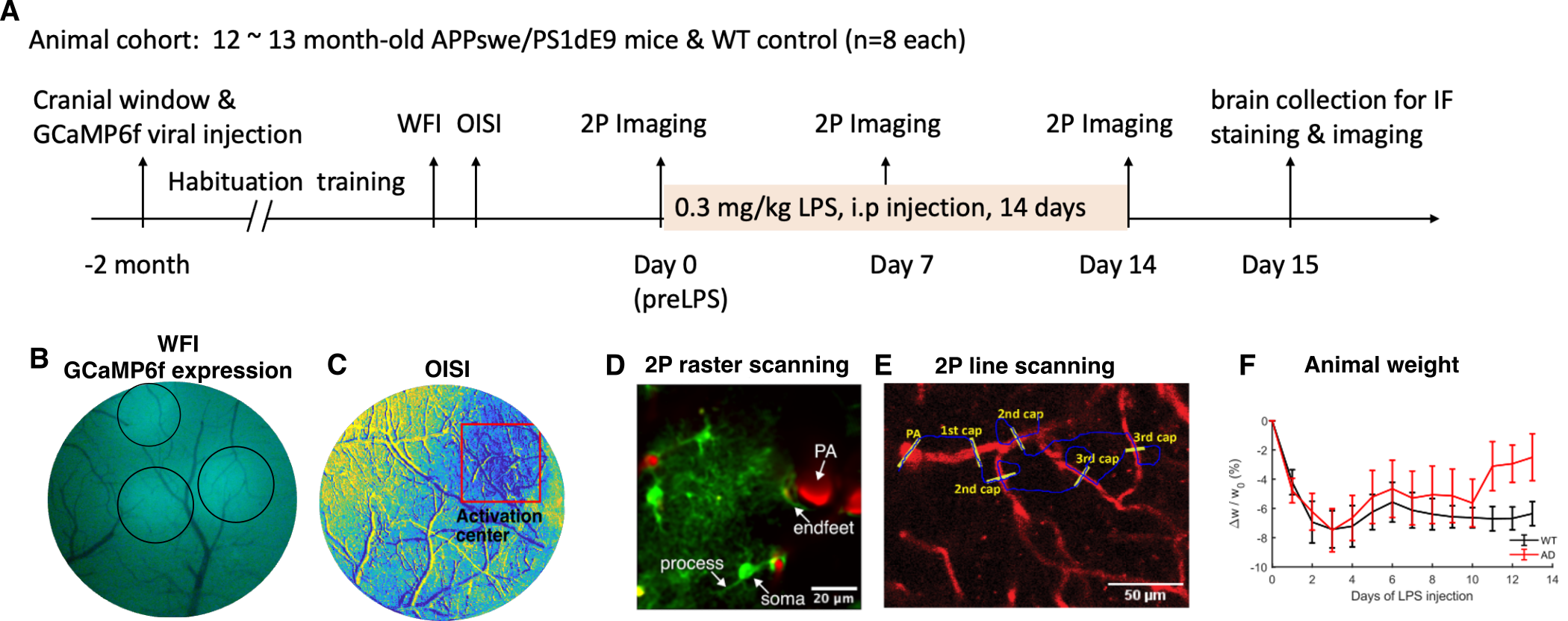


**Supplementary Figure 1.** (A) Timeline of the experimental procedure. (B) Checking of GCaMP6f virus expression using wide-field fluorescence imaging (WFI). Expression is observed in the circled regions. (C) Optical intrinsic signal imaging (OISI) mapping of the activation center during whisker stimulation. (D) Two-photon raster-scan imaging of penetrating arteriole dilation and astrocyte Ca^2+^ activity. (E) Two-photon line-scan imaging of capillary diameter and red blood cell velocity. (F) Relative weight of the animals during 14 days of LPS injection.


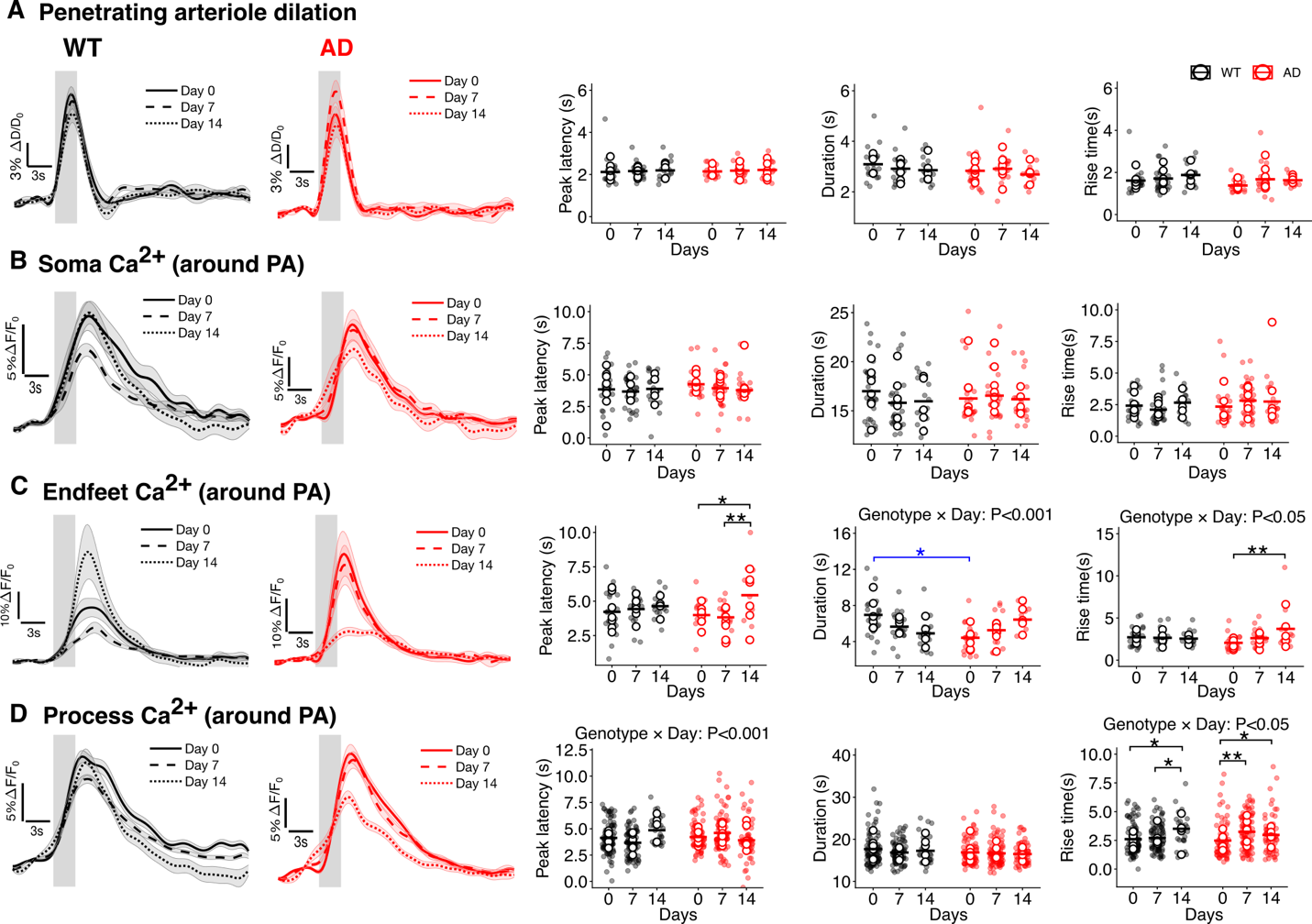
**Supplementary Figure 2.** Effects of LPS on penetrating arteriole dilation and astrocytic Ca²⁺ responses to 3 s sensory stimulation in the barrel cortex of WT and AD mice, with quantification of response kinetics (peak latency, duration, and rise time). Differences between WT and AD were assessed using a linear mixed-effects model with genotype as a fixed effect and animal as a random effect. Effects of LPS on response kinetics were tested using a linear mixed-effects model with time and genotype as fixed effects and animal as a random effect. **Blue asterisks indicate the LPS-induced changes on different days of measurement.**


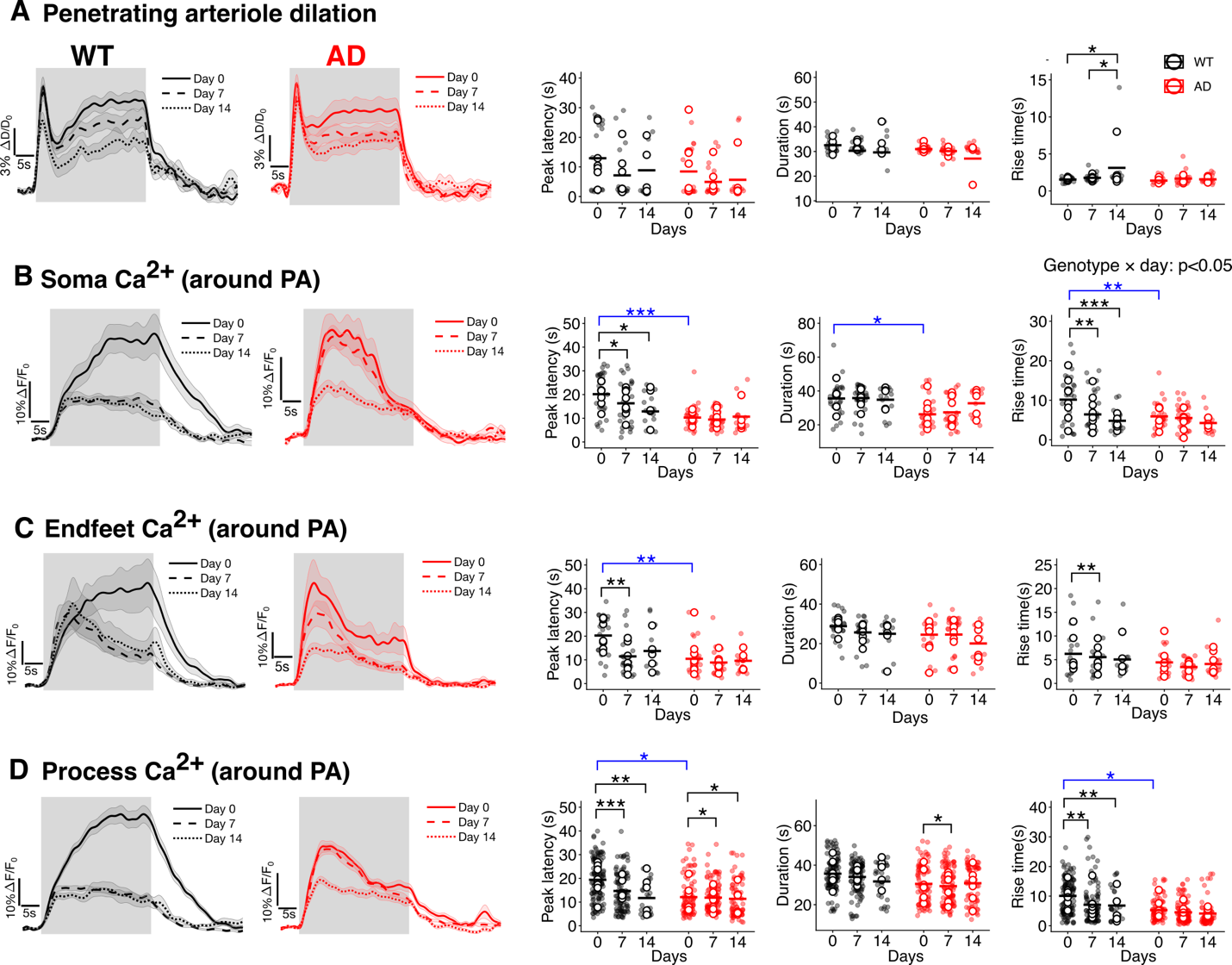


**Supplementary Figure 3.** Effects of LPS on penetrating arteriole dilation and astrocytic Ca²⁺ responses to 30 s sensory stimulation in the barrel cortex of WT and AD mice, with quantification of response kinetics (peak latency, duration, and rise time). Differences between WT and AD were assessed using a linear mixed-effects model with genotype as a fixed effect and animal as a random effect. Effects of LPS on response kinetics were tested using a linear mixed-effects model with time and genotype as fixed effects and animal as a random effect. **Blue asterisks indicate the difference between WT and AD on day 0 (pre-LPS measurements).**


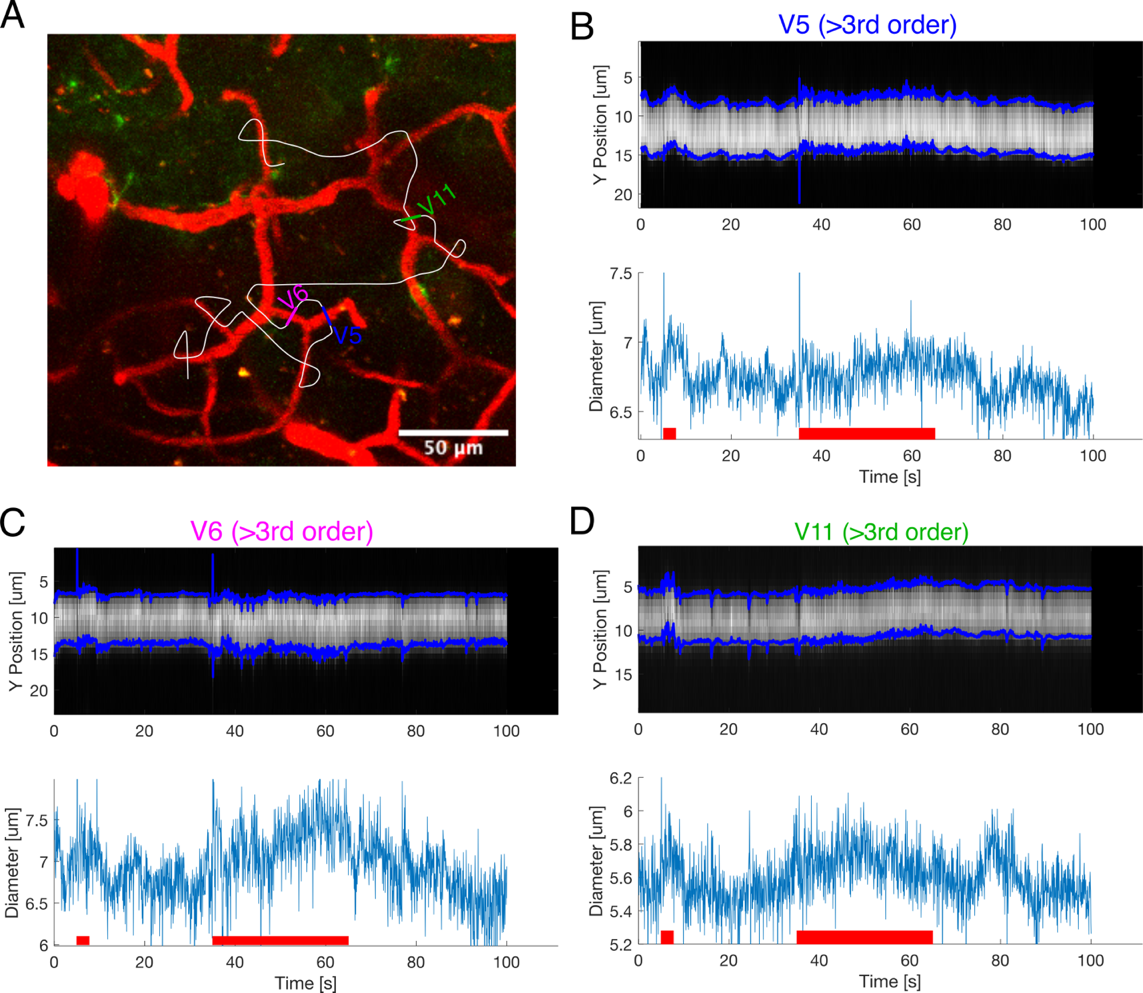


**Supplementary Figure 4. Two-photon line-scan measurements of capillary dilation during whisker stimulation.** (A) Representative image showing capillary segments (V5, V6, V11) in the barrel cortex. Image was a maximum intensity projection with a thickness of 45 μm. The scan trajectory is overlaid on the vascular image. (B–D) Time-resolved diameter changes of capillaries V5, V6, and V11 during 3 s and 30 s whisker stimulation. Upper panels show space–time line-scan images with vessel boundaries overlaid (blue), detected using the MATLAB toolbox CHIPS, and lower panels show the corresponding diameter traces. Red bars indicate the stimulation periods.


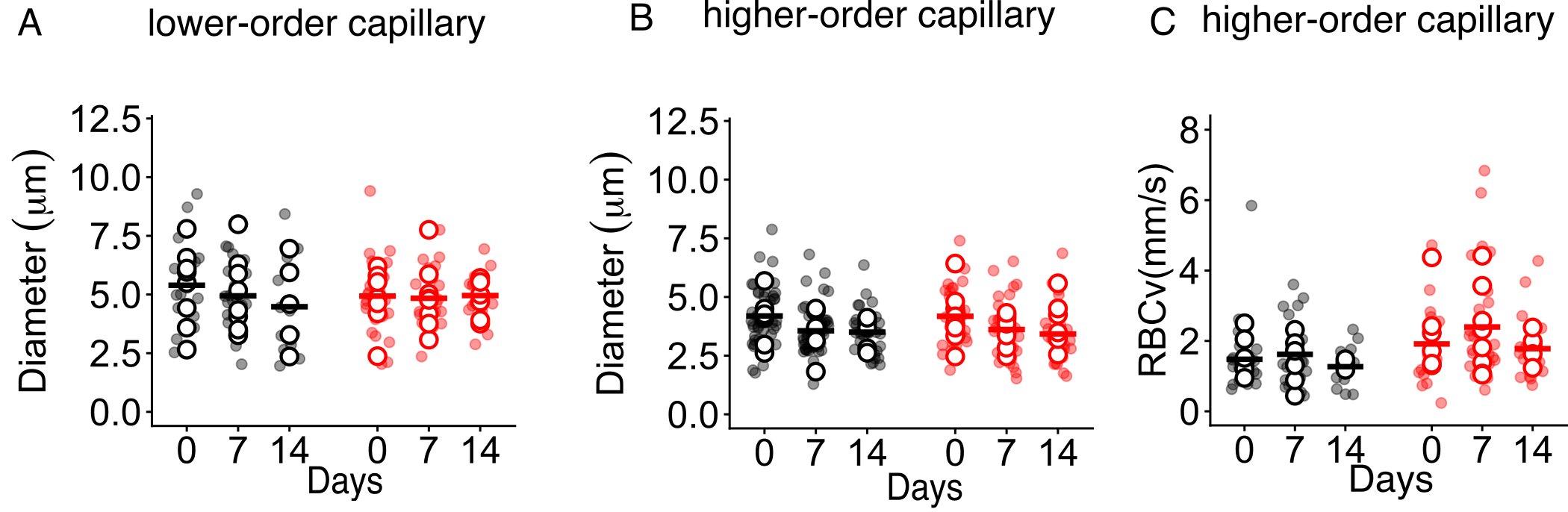


**Supplementary Figure 5.** Resting-state capillary diameter and RBC velocity under 14 days of systemic inflammatory threat. **(A)** Diameter of lower-order capillary in WT and AD mice. **(B)** Diameter of higher–order capillary in WT and AD mice. **(C)** RBC velocity in higher–order capillaries in WT and AD mic. No statistical significance was detected by linear mixed effect model.


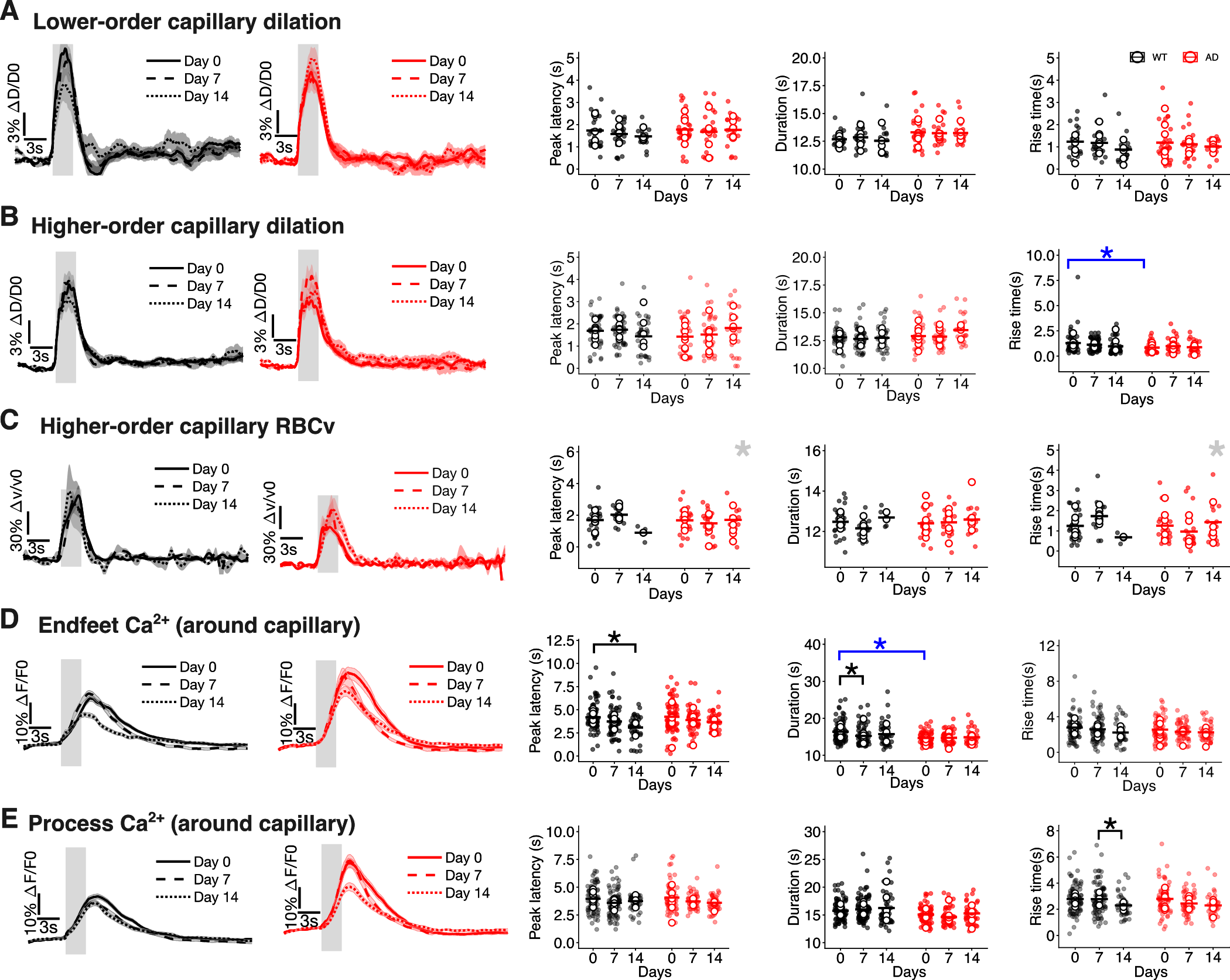
**Supplementary Figure 6.** Effects of LPS on capillary and astrocytic Ca^2+^ responses to 3 s sensory stimulation in the barrel cortex of WT and AD mice, with quantification of response kinetics (peak latency, duration, and rise time). Differences between WT and AD were assessed using a linear mixed-effects model with genotype as a fixed effect and animal as a random effect. Effects of LPS on response kinetics were tested using a linear mixed-effects model with time and genotype as fixed effects and animal as a random effect. **Blue asterisks indicate the difference between WT and AD on day 0 (pre-LPS measurements).**


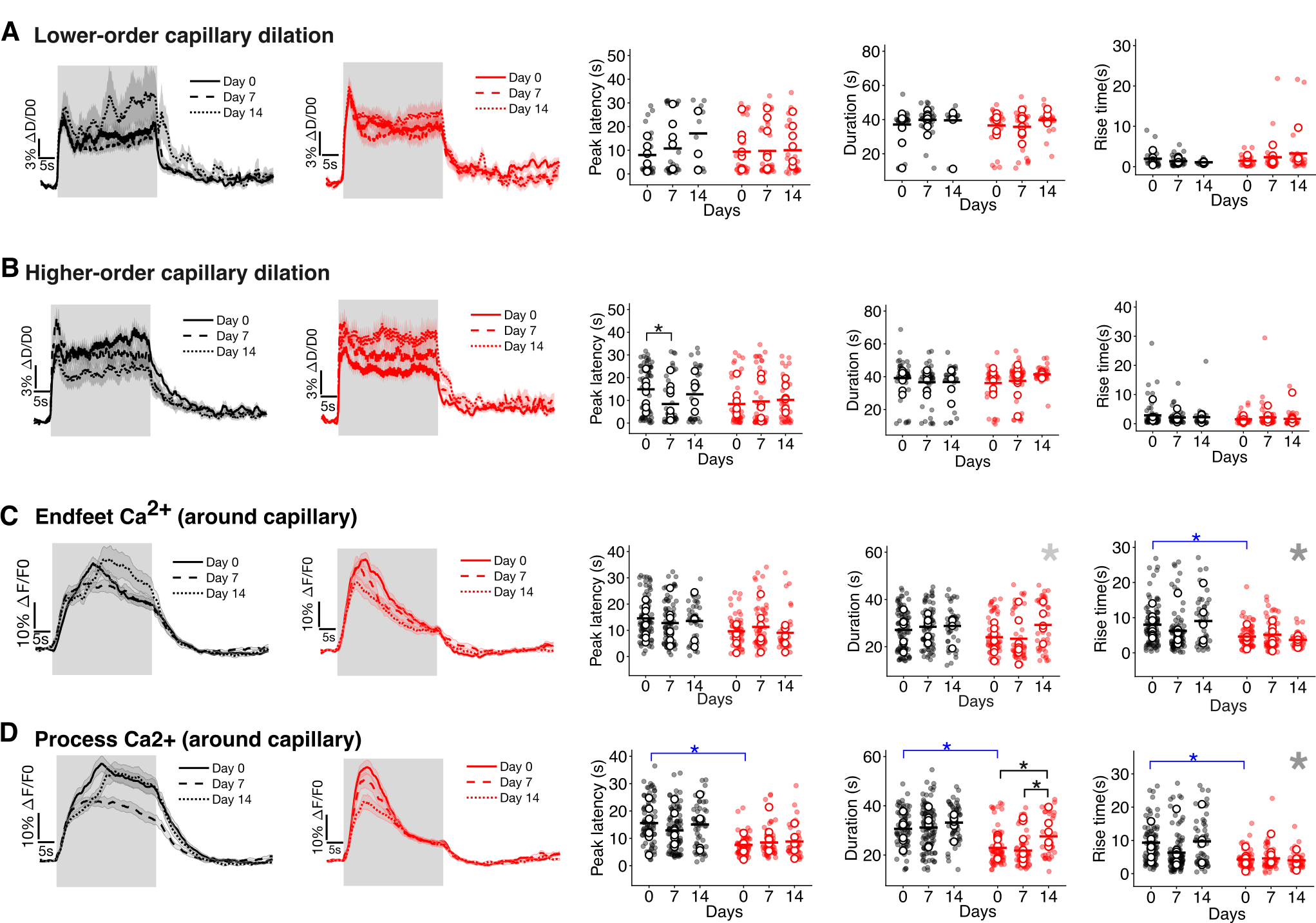


**Supplementary Figure 7.** Effects of LPS on capillary and astrocytic Ca^2+^ responses to 30 s sensory stimulation in the barrel cortex of WT and AD mice, with quantification of response kinetics (peak latency, duration, and rise time). Differences between WT and AD were assessed using a linear mixed-effects model with genotype as a fixed effect and animal as a random effect. Effects of LPS on response kinetics were tested using a linear mixed-effects model with time and genotype as fixed effects and animal as a random effect. **Blue asterisks indicate the difference between WT and AD on day 0 (pre-LPS measurements).**

**Supplementary Figure 8. Ex vivo immunofluorescence imaging.** (A) Representative confocal immunofluorescence image of GFAP and Iba-1expression in barrel cortex and hippocampus in WT and AD mice. Scale bar = 100 mm. (B) Quantification of GFAP and IBA-1 expression in barrel cortex and hippocampus. (C) Immunofluorescence image of $A\beta_{1-42}$ in AD and WT mice that received 14 days of saline injection, 7 days of LPS injection and 14 days of LPS injection. (D) Quantification of $A\beta_{1-42}$ in cerebral cortex and hippocampus. Multiple ROIs were quantified and averaged for each animal (percentage area, mean $\pm$ sem). One-way ANOVA with Tukey’s post hoc test was used in (B) and (D) to test the difference between saline, 7 days LPS and 14 days LPS injection. Differences between WT and AD saline control group in (B) and (D) were tested using Student’s t test. *p<0.05, **p<0.01, ***p<0.001.

Table 1. Sample sizes (n=ROIs, N=animals) for spontaneous astrocyte Ca^2+^ activity.

|  | | WT(n/N) | | | AD (n/N) | | |
| --- | --- | --- | --- | --- | --- | --- | --- |
| ROI | Day 0 | | Day 7 | Day 14 | Day 0 | Day 7 | Day 14 |
| Soma | 25/8 | | 23/8 | 12/4 | 30/8 | 22/7 | 16/5 |
| Process | 156/8 | | 111/8 | 46/6 | 101/8 | 103/8 | 68/7 |
| Endfeet | 14/7 | | 17/8 | 8/6 | 12/8 | 11/7 | 8/5 |

Table 2. Number of ROIs (n) and animals (N) for two-photon line-scan measurement of capillary diameter, RBC velocity, and Ca^2+^ release in astrocytic endfeet and processes around the capillary bed during 3s whisker stimulation.

|  | WT (n/N) | | | AD (n/N) | | |
| --- | --- | --- | --- | --- | --- | --- |
| ROI | Day 0 | Day 7 | Day 14 | Day 0 | Day 7 | Day 14 |
| lower-order capillary dilation | 22/8 | 23/8 | 14/5 | 26/8 | 22/8 | 18/6 |
| Higher-order capillary dilation | 46/8 | 49/7 | 14/5 | 30/8 | 34/8 | 26/6 |
| RBCv in higher-order capillaries | 24/8 | 12/7 | 4/2 | 28/7 | 18/7 | 16/6 |
| Endfeet | 92/8 | 69/8 | 39/5 | 71/7 | 55/7 | 42/5 |
| Process | 83/8 | 86/7 | 42/5 | 63/7 | 46/5 | 38/5 |

Table 3. Number of ROIs (n) and animals (N) for two-photon line-scan measurement of capillary diameter, RBC velocity, and Ca^2+^ release in astrocytic endfeet and processes around the capillary bed during 30s whisker stimulation.

|  | WT (n/N) | | | AD (n/N) | | |
| --- | --- | --- | --- | --- | --- | --- |
| ROI | Day 0 | Day 7 | Day 14 | Day 0 | Day 7 | Day 14 |
| Lower-order capillary dilation | 22/8 | 32/7 | 11/4 | 27/7 | 29/8 | 26/6 |
| Higher-order capillary dilation | 59/8 | 47/8 | 27/5 | 37/8 | 49/8 | 36/6 |
| RBCv in higher-order capillaries | 28/7 | 20/7 | 12/4 | 18/6 | 30/8 | 17/5 |
| Endfeet | 92/8 | 79/8 | 39/5 | 67/7 | 55/7 | 38/5 |
| Process | 79/7 | 94/8 | 53/5 | 66/7 | 55/7 | 37/5 |

Table 4. Number of ROIs (n) and animals (N) for two-photon raster-scan measurement of penetrating arteriole diameter (PA), and astrocyte Ca^2+^ during 3s whisker stimulation.

|  | WT (n/N) | | | AD (n/N) | | |
| --- | --- | --- | --- | --- | --- | --- |
| ROI | Day 0 | Day 7 | Day 14 | Day 0 | Day 7 | Day 14 |
| PA | 22/7 | 22/8 | 15/5 | 19/8 | 17/8 | 13/6 |
| Soma | 23/8 | 29/7 | 15/5 | 21/7 | 28/7 | 19/5 |
| Process | 88/8 | 86/8 | 25/5 | 77/7 | 86/8 | 62/6 |
| Endfeet | 20/7 | 15/6 | 15/5 | 18/8 | 16/8 | 10/6 |

Table 5. Number of ROIs (n) and animals (N) for two-photon raster-scan measurement of penetrating arteriole diameter (PA), and astrocyte Ca^2+^ during 30s whisker stimulation

| ROI | WT (n/N) | | | AD (n/N) | | |
| --- | --- | --- | --- | --- | --- | --- |
|  | Day 0 | Day 7 | Day 14 | Day 0 | Day 7 | Day 14 |
| PA | 18/7 | 18/8 | 9/5 | 18/8 | 19/8 | 14/6 |
| Soma | 26/8 | 28/8 | 14/5 | 24/7 | 32/8 | 12/5 |
| Process | 114/8 | 98/8 | 24/5 | 94/7 | 114/8 | 71/6 |
| Endfeet | 19/7 | 18/8 | 11/5 | 18/8 | 19/8 | 13/6 |
